# Analysis of the structural quality of the CASD-NMR 2013 entries

**DOI:** 10.1007/s10858-015-9949-0

**Published:** 2015-06-03

**Authors:** Timothy J. Ragan, Rasmus H. Fogh, Roberto Tejero, Wim Vranken, Gaetano T. Montelione, Antonio Rosato, Geerten W. Vuister

**Affiliations:** Department of Biochemistry, School of Biological Sciences, University of Leicester, Henry Wellcome Building, Lancaster Road, Leicester, LE1 9HN UK; Departamento de Química Física, Universidad de Valencia, Avda. Dr. Moliner 50, 46100 Burjassot (Valencia), Spain; Structural Biology Brussels, Vrije Universiteit Brussel, Pleinlaan 2, Brussels, Belgium; (IB)2 Interuniversity Institute of Bioinformatics in Brussels, ULB-VUB, Triomflaan, 1050 Brussels, Belgium; Center for Advanced Biotechnology and Medicine, Department of Molecular Biology and Biochemistry, and Northeast Structural Genomics Consortium, Rutgers, The State University of New Jersey, Piscataway, NJ 08854 USA; Robert Wood Johnson Medical School, Piscataway, NJ 08854 USA; Magnetic Resonance Center, Department of Chemistry, University of Florence, 50019 Sesto Fiorentino, Italy

**Keywords:** Protein, NMR, Structure determination, Quality, Validation, Blind testing, NOE, CASD-NMR

## Abstract

We performed a comprehensive structure validation of both automated and manually generated structures of the 10 targets of the CASD-NMR-2013 effort. We established that automated structure determination protocols are capable of reliably producing structures of comparable accuracy and quality to those generated by a skilled researcher, at least for small, single domain proteins such as the ten targets tested. The most robust results appear to be obtained when NOESY peak lists are used either as the primary input data or to augment chemical shift data without the need to manually filter such lists. A detailed analysis of the long-range NOE restraints generated by the different programs from the same data showed a surprisingly low degree of overlap. Additionally, we found that there was no significant correlation between the extent of the NOE restraint overlap and the accuracy of the structure. This result was surprising given the importance of NOE data in producing good quality structures. We suggest that this could be explained by the information redundancy present in NOEs between atoms contained within a fixed covalent network.

**Electronic supplementary material:**

The online version of this article (doi:10.1007/s10858-015-9949-0) contains supplementary material, which is available to authorized users.

## Introduction

In the CASD-NMR-2013 effort (see accompanying paper, Rosato et al. 2015), 164 entries were submitted across ten targets. Together, these data provide for the opportunity to assess the current state of automated structure calculation methods for small- to medium-sized proteins. Automated methods (summarized in Donald and Martin 2009; Gossert et al. 2011; Guerry and Herrmann 2011; Güntert and Buchner 2015; Herrmann et al. 2002; Huang et al. 2006; Linge et al. 2003a; Williamson and Craven 2009) greatly speed up the process of NMR structure determination by providing an alternative to the manual, labor intensive step of NOESY peak assignment. In addition, it is to be expected that these procedures also provide a more unbiased interpretation of the available data. Some automated methods are even purely chemical-shift (CS) based (Shen et al. 2008), thus requiring no additional data at all and further reducing the required experimental time and associated costs in terms of equipment and labor.

For any structure, whether derived by automated methods or manually, it is imperative that the result is accurate and properly reflects the underpinning data. Ultimately, it is the aim to use the structure to explain biology, either by the researchers that generated them or indirectly by others through deposition in the PDB archive. Prompted by instances of serious errors and allegations of fabricated data underpinning published structures the wwPDB, as curator of the PDB archive, initiated a number of policy changes to improve its quality and integrity. Consequently, it is now mandatory to deposit experimental NMR restraints and assigned NMR chemical shifts. In addition, an expert NMR validation task force (NMR-VTF) has published a set of recommendations for validating NMR-derived structures and accompanying experimental data (Montelione et al. 2013).

In the following, we present a comprehensive validation report on the entries and targets of CASD-NMR-2013 in line with the NMR-VTF recommendations. The analysis draws upon the tools for validating geometric quality in relation to known structural data and the assessment of structural quality in relation to the experimental data. We used commonly available program suites CING (Doreleijers et al. 2012a), Molprobity (Lovell et al. 2003) and PSVS (Bhattacharya et al. 2006). By validating all of the entries in the same way, we are able to show the strengths and weaknesses of the various automated structure generation programs and assess the complementarity of these structure validation tools. In addition, we are able to draw broader conclusions across the range of automated structure generation routines participating in the CASD-NMR-2013 effort.

## Methods

The CASD-NMR-2013 entries and their metadata were downloaded from the WeNMR (Wassenaar et al. 2012) web site whereas the targets were obtained from the BMRB and RCSB wwPDB repositories. We adopt the definitions of target and entry given in the accompanying paper describing the CASD-NMR-2013 data (see Table 1 and the accompanying paper, Rosato et al. 2015), where the target comprises all originating data, the manually derived restraints and resulting structural ensemble. An entry denotes an ensemble of conformers and the accompanying restraints generated by a specific program for a specific target.

**Table 1:** CASD-2013 targets

Target ID	PDB ID	Valid range(s)	Reference ensemble authors
HR2876B	2LTM	13–105	Liu, G., Xiao, R., Janjua, H., Hamilton, K., Shastry, R., Kohan, E., Acton, T.B., Everett, J.K., Lee, H., Huang, Y.J., Montelione, G.T.
HR2876C	2M5O	17–91	Liu, G., Xiao, R., Janjua, H., Hamilton, K., Shastry, R., Kohan, E., Acton, T.B., Everett, J.K., Pederson, K., Huang, Y.J., Montelione, G.T.
HR5460A	2LAH	14–25, 33–158	Liu, G., Shastry, R., Ciccosanti, C., Hamilton, K., Acton, T.B., Xiao, R., Everett, J.K., Montelione, G.T.
HR6430A	2LA6	14–99	Liu, G., Xiao, R., Janjua, H., Lee, H., Ciccosanti, C.T., Acton, T.B., Everett, J.K., Huang, Y.J., Montelione, G.T.
HR6470A	2L9R	554–608	Liu, G., Xiao, R., Lee, H.-W., Hamilton, K., Ciccosanti, C., Wang, H.B., Acton, T.B., Everett, J.K., Huang, Y.J., Montelione, G.T.
HR8254A	2M2E	15–56	Lemak, A., Yee, A., Houliston, S., Garcia, M., Ong, M., Arrowsmith, C.
OR135	2LN3	4–74	Liu, G., Koga, R., Koga, N., Xiao, R., Lee, H., Janjua, H., Kohan, E., Acton, T.B., Everett, J.K., Baker, D., Montelione, G.T.
OR36	2LCI	2–46, 53–125	Liu, G., Koga, N., Koga, R., Xiao, R., Lee, H.T., Janjua, H., Ciccosanti, C., Acton, T.B., Everett, J., Baker, D., Montelione, G.T.
StT322	2LOJ	23–63	Wu, B., Yee, A., Houliston, S., Garcia, M., Savchenko, A., Arrowsmith, C.H.
YR313A	2LTL	17–41, 45–115	Liu, G., Xiao, R., Hamilton, K., Janjua, H., Shastry, R., Kohan, E., Acton, T.B., Everett, J.K., Lee, H., Huang, Y.J., Montelione, G.T.

The PDB ID, valid ranges and reference ensemble sources for comparison of each target is given

The data sets were reorganized into a uniform directory structure to allow processing by the software analysis pipeline. Ambiguous header data and missing and damaged files were queried with the depositors, and errors discovered during processing, such as incorrect file formats, unsupported naming conventions or atom name errors in the structure files, etc., were corrected. Structure ensembles were read into CcpNmr Analysis 2.4 from PDB-type files using the Analysis structure reader (built on CcpNmr FormatConverter parsers) (Vranken et al. 2005), which disambiguated the varied naming conventions employed, and reported errors for correction. The deposited sequences were aligned automatically with those read from the target data to identify truncations. Restraint files were read into the same CCPN project using CcpNmr FormatConverter in automatic mode to identify, classify, read, and integrate the restraint files for each submission. The resulting CCPN projects each contained all data for a single target or entry, grouped so that they could be automatically extracted by CING for analysis. Due to technical limitations we were not able to incorporate data from the so-called ARIA ‘swap files’ that describe conformer-specific stereospecific resonance assignments. Accordingly, we were forced to reduce the precision of the deposited restraints to non-stereospecific for the one program that uses different assignments of prochiral groups in each individual structure of the ensemble.

Entries were assigned three-part names with each part separated by an underscore. The first part of the name is the target dataset. The second part is the program used in generating the entry, merging CS-ROSETTA and CS-DP-ROSETTA together as ‘Web Rosetta Server’; CS-HM-ROSETTA and CS-HM-DP-ROSETTA as ‘CS-HM-ROSETTA and Cheshire and Cheshire-YAPP as ‘Cheshire’. The last part of the name describes what input data were used. The first character indicates curated NOE peaks (c), un-curated NOE peaks (u), raw spectra (r), and CS only (s); if RDCs were used, ‘r’ has been appended to the end of the data identifier. Finally, if the input sequence was truncated manually, the truncated range used is indicated in parenthesis. Using this merging strategy, no information is lost—for example Cheshire uses only CS data and entries are listed as ‘Cheshire_s’, while Cheshire-YAPP uses both CS and NOE data and entries are listed as either ‘Cheshire_c’ or ‘Cheshire_u’.

All analyses were conducted using CING (Doreleijers et al. 2012a), except where noted. CING integrates the results of a number of external programs, such as WHAT-IF (version 6) (Vriend 1990), PROCHECK_NMR (Laskowski et al. 1996), Wattos (Doreleijers et al. 2009) and VASCO (Rieping and Vranken 2010), combined with its own internal routines. All analyses were conducted on residues within the well-defined areas of the reference ensembles as determined by CyRange (Table 1) (Kirchner and Güntert 2011). The analysis of Discriminating Power (DP) scores (Huang et al. 2005) and number of atomic clashes was performed using the PSVS (Bhattacharya et al. 2006) server (http://psvs-1_5-dev.nesg.org/). PSVS integrates analyses from several widely-used structure quality evaluation tools, including RPF (Huang et al. 2005), PROCHECK (Laskowski et al. 1993), PROCHECK_NMR (Laskowski et al. 1996), Ramachandran (Lovell et al. 2003), Verify3D (Lüthy et al. 1992), Prosa II (Sippl 1993) and probe (Word et al. 1999). For the DP score determination the (curated) NMR peak lists and chemical shifts from the targets were used. The number of clashes was obtained as the number of disallowed atom pair overlaps ≥0.4 Å given by the probe (Word et al. 1999) standalone program.

All-by-all RMSD values are calculated as follows. For each of the M conformers in the query ensemble, the RMSD between the backbone N, Cα and C’ atoms in the well-defined region of the reference ensemble as defined by CyRange (Kirchner and Guentert 2011) (see Table 1) of each of the N conformers in the target ensemble is calculated, yielding a list of M × N RMSD values (or $\frac{{{\text{M}}({\text{M}} - 1)}}{2}$ values for convergence calculations where the same ensemble is both the query and target). If an entry is lacking any atoms within the well-defined range, the corresponding atoms in the compared ensemble are ignored. The average value of this list of values is then reported as the mean RMSD. The accuracy of an ensemble is defined as the all-by-all RMSD of an entry to the appropriate target ensemble. Ensemble convergence values are reported as the average all-by-all RMSD of the conformers in an ensemble. The ensemble convergence calculation is rapid and independent of both the nature of the experimental input data and the structure determination algorithm method used and should not be confused with ensemble precision. Ensemble convergence often underreports the actual precision of an ensemble, as prior research showed that ensembles with a much larger RMSD could be generated that equally well satisfied the experimental restraints (Buchner and Güntert 2015; Spronk et al. 2003). Accordingly, ensemble convergence is used here as a diagnostic criterion only.

NOE overlap values were calculated using a custom Python script, available from the authors on request. Each value was calculated as follows: each NOE in the query list (row in Fig. 5c, d) was compared to each NOE in the subject list (column in Fig. 5c, d) until either a match was found or there were no more NOEs in the subject list. To ensure ambiguous restraints were counted only once, the search was terminated once a match was found to any of the options. Note that handling ambiguous restraints in this way has the side effect that multiple ambiguous restraints in the query list can match a single restraint in the subject list. Heatmaps of all restraint overlaps for all ten targets are shown in the Supplementary materials.

Supplementary Table 1 lists entry and validation statistics of all 169 entries, including for reference also six entries marked ‘incorrect’ by the depositing authors. The CING validation reports and csv files of all the accumulated data, including restraint violation statistics and all values underpinning the figures in this manuscript, are available from our website http://nmr.le.ac.uk/CASD-NMR-2013. A PostgreSQL database containing the complete CING analysis for all targets and entries in CASD-NMR-2013 is available from the authors upon request.

## Results

### Accuracy and ensemble convergence

**Fig. 1:**
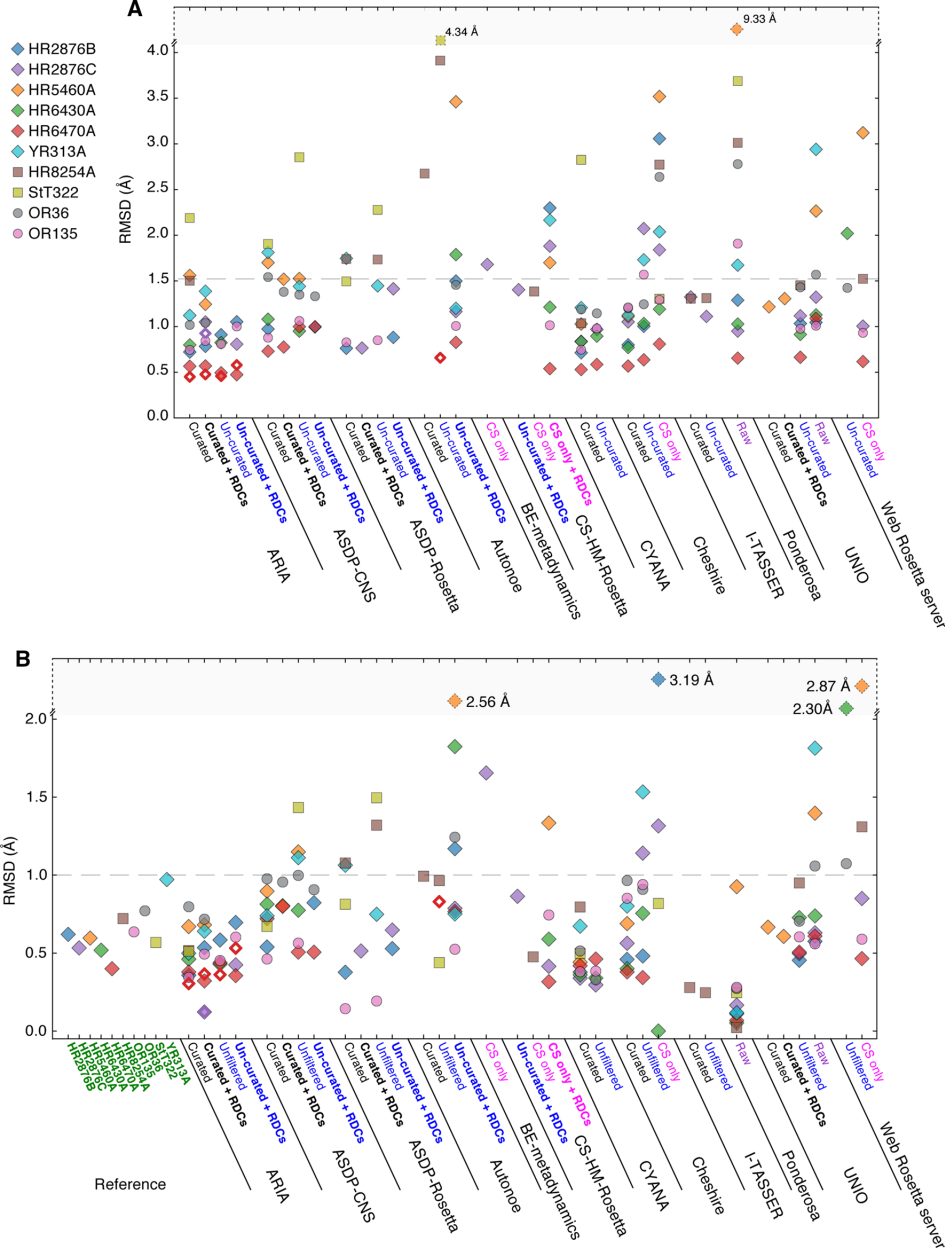
Comparison of targets and entries. **a** Structural similarity (accuracy): the mean all versus all pairwise backbone RMSD for well-defined residues for each of the entries with respect to the target. The *dashed line* at 1.5 Å indicates a reasonable upper threshold for identity within experimental uncertainty (see text for details). **b** The pairwise backbone RMSD for well-defined residues within each ensemble for each of the targets and entries. The *dashed line* at 1.0 Å indicates an estimated upper threshold for a converged structure. *Symbols* for each target are indicated on the *left*. *Open symbols* indicate entries generated from truncated input sequences. *Horizontal axis labels*: targets are labeled in *green*, entries generated from curated lists in *black*, curated lists plus RDCs in *bold-black*, un-curated lists in *blue*, un-curated lists plus RDCs in *bold-blue*, CS only in *magenta*, CS plus RDCs in *bold magenta* and raw data in *purple*

The ensemble convergence of each of the CASD-NMR-2013 entries and target ensembles and the similarity of the entry to the corresponding target ensemble were assessed using the deviation of the backbone coordinates, expressed as the average of the pairwise root mean square deviation (RMSD) between the conformers in the reference and entry ensembles using the well-defined regions defined for the reference ensemble by CyRange (Kirchner and Güntert 2011). For the targets, the convergence ranges from 0.4 to 1.0 Å (Fig. 1). Therefore, we consider 1.0 Å to be an appropriate threshold to identify satisfactorily converged calculations. The median convergence for the entries is 0.6 Å with 77 % of the entries having an ensemble convergence of 1.0 Å or less. Only five entries have values larger than 2 Å: three ensembles calculated from augmented CS data and two ensembles calculated from CS data only. For programs that submitted entries based on un-curated and curated NOESY peak lists, we observed a weak tendency to obtain better ensemble convergence with the curated list when the ensemble convergence for the un-curated list was above the 1 Å threshold.

In the CASD-NMR effort, the manually determined target ensemble is assumed to be the correct representation of the three-dimensional structure of the target protein. Hence, the RMSD between the target ensemble and the entry ensemble constitutes a measure of accuracy (Fig. 1a). An entry is considered to be indistinguishable from the target when the RMSD between the two ensembles is less than the sum of their ensemble convergence. Given the average ensemble convergence of 0.63 Å for the targets and 0.74 Å for the entries, a threshold of 1.5 Å appears to be reasonable. Above this threshold, any ensemble describes a structure with differences from the corresponding target beyond experimental uncertainty. Each entry was evaluated relative to the corresponding target with the exception of ensembles marked as not converged by the programs used to generate them. The median accuracy over the entire dataset is 1.14 Å, with 71 % of the entries below the 1.5 Å threshold. Approaches using curated NOESY peak lists achieved the highest accuracy with a median accuracy of 1.05 Å and 80 % of the entries below the threshold. The performance was essentially the same when un-curated NOESY peak lists were used (median accuracy 1.08 Å; 79 % of entries below the threshold). In contrast, calculations based on either raw spectral data or CS only data performed less well, with median accuracies of 1.45 and 1.52 Å, respectively, both yielding only 50 % of ensembles below the threshold.

The data collected within CASD-NMR-2013 allowed us to evaluate the dependence of the performance of automated structure generation methods on the input data, specifically comparing the use of curated NOESY peak lists relative to un-curated NOESY peak lists and/or raw spectral data. Only programs with multiple submissions using different types of input data for the same target were included in this analysis (cf. Table 2).

**Table 2:** Median accuracy of paired entries

Program	Curated (Å)	Un-curated (Å)	Raw (Å)	Number of targets
ARIA	0.78	0.91	–	5
ASDP-CNS	1.27	1.20	–	8
ASDP-ROSETTA	1.16	1.43	–	6
CHESHIRE-YAPP	1.05	1.24	–	7
CYANA	0.84	0.97	–	5
UNIO	–	1.01	1.11	6

Only targets calculated using both curated and un-curated data (or un-curated and raw data, in the case of UNIO) are included. Note that no program submitted paired entries for all targets and therefore comparison of accuracies made across programs is potentially inappropriate (see text)

Firstly, we compared the use of curated and un-curated NOESY peak lists for methods that rely predominantly on NOESY data. ARIA submitted entries for five targets that allow for such a comparison. The median accuracy is 0.91 Å for the un-curated peak lists and 0.78 Å for the curated peak lists, suggesting that the use of curated peak lists does improve the accuracy. However, it should be noted that the accuracy of each entry is well within the 1.5 Å threshold for good quality ensembles regardless whether un-curated or curated peak lists were used. Similarly, for the ten qualifying entries (for five targets) submitted by CYANA, the median accuracy for entries generated from un-curated peak lists is slightly lower at 0.97 Å when compared to the values obtained for entries generated from the curated peak lists (0.84 Å), again with the accuracy for all entries comfortably within the threshold. Overall, ASDP performed slightly less well than ARIA or CYANA (but see below). Based on entries for six targets, four ensembles generated by ASDP-Rosetta achieved the required accuracy using either un-curated or curated peak lists and the median accuracies were similar at 1.43 and 1.16 Å, respectively. Interestingly, for ASDP-CNS the proportion of entries within the accuracy cutoff rose from five out of eight generated from curated peak lists to six out of eight entries generated from un-curated peak lists, with median accuracies of 1.27 and 1.20 Å, respectively.

One deficiency in our analysis is the incomplete nature of the dataset. For example, ARIA and CYANA both submitted five paired entries, four of which were for the same targets, but of the six target pairs submitted by ASDP-Rosetta, only three pairs were also submitted by ARIA and two pairs were also submitted by CYANA (and for one pair RDC restraints are used by ASDP-Rosetta and not by CYANA). As a result, any comparisons made across programs could lead to inappropriate conclusions. Indeed, ASDP-Rosetta is the only program to submit paired entries for both of the two most challenging targets [StT322 and HR8254A (Rosato et al. 2015)], where ARIA and CYANA both failed to generate realistic converged structures from un-curated peak lists. Overall, our results would suggest that for algorithms relying primarily on NOESY data, ensembles of equivalent accuracy can be obtained regardless of whether curated or conservatively chosen un-curated peak lists are used as the input. It is worth noting that the two targets using the most liberal peak picking algorithm (i.e., StT322 and HR8254A,) and therefore including the largest fraction of probable noise peaks, proved the most difficult to solve using these fully automated analysis methods.

Cheshire-YAPP generates ensembles based on CS data then filters these ensembles based on NOESY distance restraints. Using un-curated peak lists, Cheshire-YAPP submitted entries for seven targets achieving a median accuracy of 1.24 Å with four entries within the 1.5 Å threshold. In this case, the use of curated peak lists significantly improved the accuracy of the entries as, out of the eight targets submitted, the median accuracy for the seven matched pairs improved to 1.05 Å and the accuracy of all eight entries was within the threshold.

Only one program, UNIO, submitted entries based on both peak lists (Un-curated) and raw spectral data. Entries were submitted for six targets with all of the ensembles generated based on un-curated peak lists displaying an accuracy within the 1.5 Å RMSD threshold. Similarly, five of the six entries generated from raw spectral data achieved the desired accuracy, with the sixth entry (OR36) yielding a still acceptable accuracy of 1.6 Å. A small decline in the median accuracy (from 1.01 to 1.11 Å) was observed for the UNIO entries derived from raw spectral data compared to those calculated from un-curated peak lists.

Finally, we note that all but one of the entries misidentified the only *cis*-Proline in the target set, Pro142 in HR5460A as a *trans*-proline. *Cis*-prolines are normally identified using the chemical shift difference between ^13^C^β^ and ^13^C^γ^, with 0 ppm ≤ ^13^C^β^–^13^C^γ^ ≤ 4.8 ppm strongly indicative of a *trans* conformation and 9.15 ≤ ^13^C^β^–^13^C^γ^ ≤ 14.4 strongly indicative of a *cis* conformation (Schubert 2002) and/or by characteristic sequential H^α^/H^α^ NOEs. The ^13^C^β^–^13^C^γ^ value for Pro142 in HR5460A is 7.88 ppm, hence in the transition region between these two chemical shift ranges. Only CS-HM-Rosetta successfully identified Pro142 as a *cis*-proline, but only in six out of ten conformers in the ensemble. This suggests that more rigorous determination of proline isomer state may be appropriate for all methods (including CS-HM-Rosetta).

### Geometric and packing quality

**Fig. 2:**
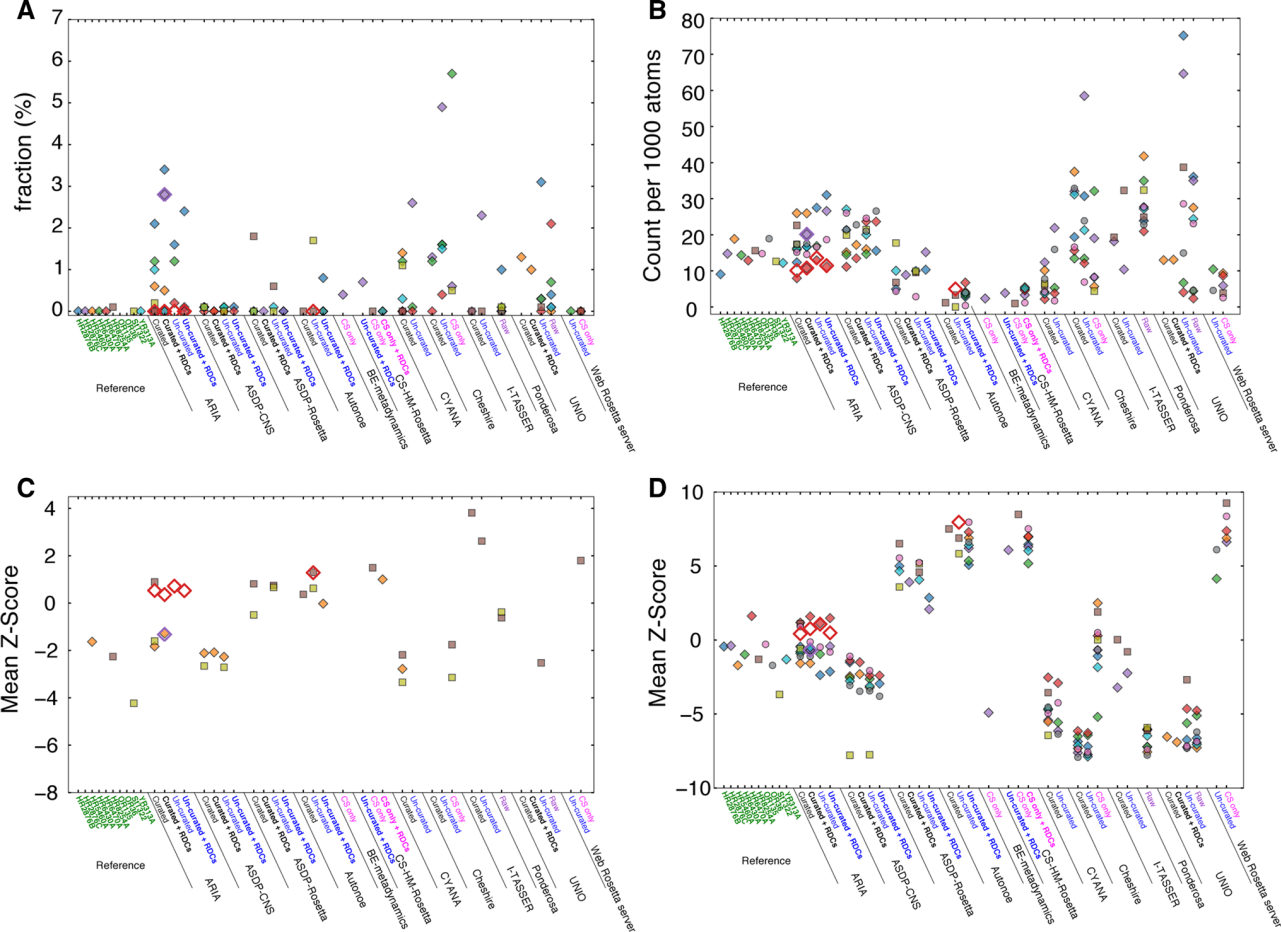
Overall quality scores of the targets and the entries. **a** Molprobity Ramachandran outliers (Lovell et al. 2003). **b** Molprobity number of clashes per thousand atoms in the ensemble. **c** WHAT-IF Ramachandran Z-scores (Vriend 1990). **d** WHAT-IF side chain *Z*-scores. *Symbols* and *labels* are explained in the legend for Fig. 1

Structures can be validated by comparison of a set of metrics relative to those obtained from reference structures. We used the scores of the programs Molprobity (Lovell et al. 2003) and WHAT-IF (Vriend 1990), as implemented in the CING framework. Figure 2 displays four such metrics, i.e. the fraction of backbone dihedrals in the Ramachandran disallowed region (Fig. 2a), the number of high energy interatomic contacts per 1000 atoms in the ensemble (Fig. 2b), the Ramachandran backbone angle distribution (Fig. 2c) and the side chain dihedral angle distribution (Fig. 2d), for both the targets and the entries. WHAT-IF values (Fig. 2c, d) are given as the mean of the values calculated for each conformer in the ensemble. The scores reveal that the targets constitute well-refined structures, with near-zero percent of Ramachandran outliers (Fig. 2a) and Ramachandran Z-scores generally larger than −2 (Fig. 2c). Relatively few clashes are observed (Fig. 2b) and WHAT-IF side-chain Z-scores (Fig. 2d) of around zero are comparable to those observed in well-refined X-ray and NMR structures.

The values observed for the different entries vary considerably and correlate to some extent with the structure generation method, i.e. the engine used to generate the entry, with some targets displaying relatively better or worse scores across all programs. The effect of the generating method is most clearly observed from the WHAT-IF side chain dihedral scores (Fig. 2d). Structures from the Rosetta web server, CS-HM-Rosetta, Autonoe (also Rosetta-based) and ASDP-Rosetta, all display excellent median Z-scores. Conversely, CYANA, UNIO, Ponderosa, and Cheshire-YAPP have scores of around −5, whereas the other entries are intermediate between these two extremes. The excellent scores for the Rosetta-based protocols is no surprise, as its conformational sampling engine draws upon structural reference data similar to those used to generate the WHAT-IF side chain dihedral score. Low scores are indicative of non-optimal local geometry and do not imply errors in the overall fold.

The quality of the backbone geometry is expressed by the MolProbity fraction of Ramachandran outliers (Fig. 2a) and the WHAT-IF Ramachandran Z-Score (Fig. 2c). These two scores are complementary, as the MolProbity score reports the fraction of residues having nearly impossible dihedral angles while the WHAT-IF score reports the overall dihedral distribution. The entries generated by curated or un-curated Cheshire and UNIO display the poorest scores, with Cheshire also showing a large variability of the outliers scores within its submitted entries. ARIA also shows a substantial number of entries with larger outlier percentages, yet the WHAT-IF Ramachandran Z-scores are often better than those of the targets. The three Rosetta-based protocols (Autonoe, CS-HM-Rosetta and Web server Rosetta) and Ponderosa are generally good according to these two criteria, with nearly all entries displaying only small fractions of outliers and generally better Z-scores than the targets. Finally, the scores of ASDP-CNS and CYANA entries appear en-par with those of the targets.

Figure 2b shows the number of high-energy interatomic interactions, per 1000 residues, as determined by MolProbity. Here, the three Rosetta-based protocols, CYANA, and ASDP-Rosetta display the best values, below ten clashes per thousand atoms. ARIA, ASDP-CNS and Cheshire have values around twenty clashes per 1000 atoms, and UNIO, Ponderosa, and Cheshire-YAPP have median clash scores somewhat higher than the targets, with values of thirty or higher and extending up above forty. Close examination of the scores obtained for individual targets across the different entries reveals that entries for HR6470A and OR135 tend to be among the best scoring, whereas entries for StT322, HR2876C and OR36 are among the worst.

**Fig. 3:**
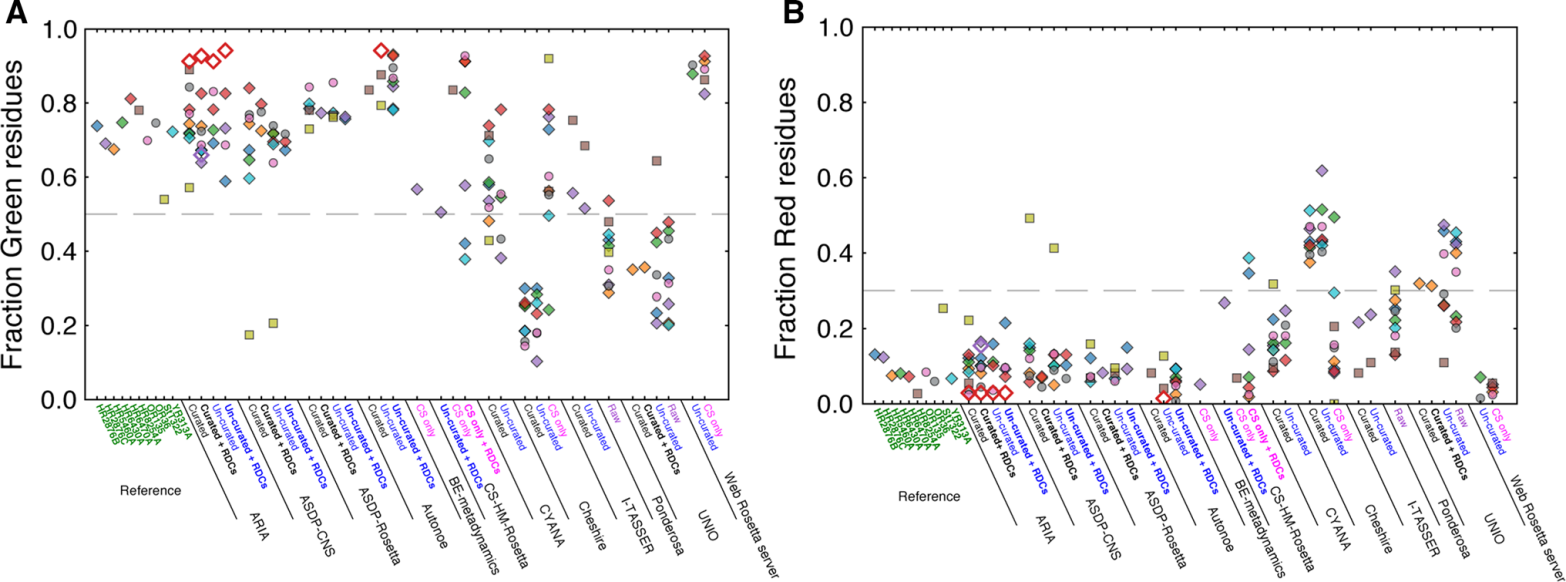
ROG scores (Doreleijers et al. 2012a) of the targets and the entries. **a** The fraction of residues with a *green* ROG score. The *lower* threshold of 0.5 is indicated by a *dashed line*. **b** The fraction of residues with a *red* ROG score. The *upper* threshold of 0.3 is indicated by a *dashed line*. *Symbols* and *labels* are explained in the legend for Fig. 1

In addition to overall validation scores as provided by programs such as MolProbity and WHAT-IF, it is also advantageous to examine residue-specific validation criteria. The CING program suite (Doreleijers et al. 2012a) implements such a residue-specific score as the so-called ROG-score. The ROG (red–orange–green) score represents a compounded measure of confidence for an individual entity, such as a residue, expressed as red for potentially problematic, orange for suspect and green for likely correct. The residue ROG score includes an assessment of the Ramachandran quality, the Omega dihedral and side chain dihedrals. The criteria are detailed in Table 2 of Doreleijers et al. (2012a). ROG-scores reported in the current paper represent the fractions of the total number of residues in the well-defined range (as identified by CyRange), with a specific red or green classification. As a rule of thumb, the number of green residues should exceed 50 % while the number of red residues should be below 30 %. Figure 3a, b display the green and red ROG-scores, respectively, for all entries and targets. In line with the overall scores, the data show that the targets display generally very good ROG scores, with all of the targets scoring better than the 50/30 % criteria. For the entries the results are more diverse. As expected, and as observed before (Rosato et al. 2012), the CS-only based methods display very good ROG scores. Good scores were also obtained for many of the NOE/RDC based protocols, e.g. ARIA, ASDP-CNS, ASDP-Rosetta, Autonoe and most of the CYANA entries. In contrast, Cheshire using peak lists, Ponderosa and UNIO score substantially worse. StT322 appears to be a problematic target, which has the worst scores of all the reference structures and consistently scores poorly for the entries as well. Most automated methods had their poorest performance with the filtered peak list for StT322, and did not even provide a submission for this target with un-curated peak lists. For methods which did provide results using the un-curated or raw StT322 data, including ASDP-CNS, ASDP-Rosetta and Ponderosa, the resulting structures were clear outliers, with low accuracies. Potentially, this target has some special features, e.g. related to either the distribution of the chemical shifts, the quality of the data or the occurrence of conformational equilibria in solution, that distinguish it from the other targets.

### Agreement with experimental data

**Fig. 4:**
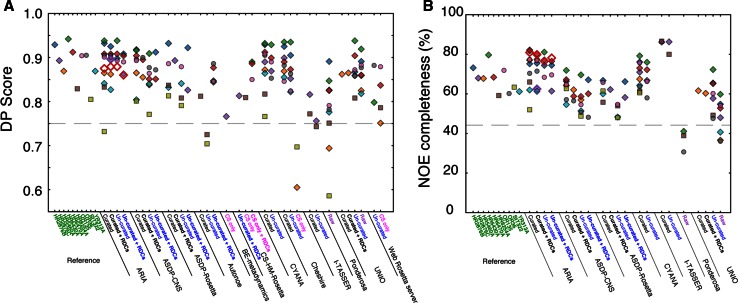
Agreement with experimental data of the targets and the entries. **a** The DP score (Huang et al. 2005). The *dashed line* indicates the lower threshold of 0.75 for agreement between the structure and the input data. **b** The NOE completeness determined by Wattos. The *dashed line* indicates the median NOE completeness (44.2 %) for all structures in the NRG-CING database (Doreleijers et al. 2012b). *Symbols* and *labels* are explained in the legend for Fig. 1. Only entries calculated from NOESY lists have been included

The completeness of the experimental data and its agreement with the ensemble of conformers constitutes another class of useful metrics to assess the structural results. The quality of the structure produced by any given method is expected to depend on the amount of experimental data, i.e. to a large extent the number of correct NOEs that can be assigned and their information content. During the evaluation of the CASD-NMR-2010 round the discriminating power (DP) score was used as a measure of the goodness-of-fit of the unassigned NOESY peak lists to the obtained structures. The DP score compares the unassigned NOESY peak lists to the generated structure, using the improvement in fit relative to a random coil reference structure to evaluate the structure quality. With possible values of 0–1, a DP lower score cutoff of ~0.7 has been considered to represent a reliable structure (Huang et al. 2012). For this round, the DP scores of the targets and the different entries are shown in Fig. 4a. As in the CASD-NMR-2010 results (Rosato et al. 2012), a correlation was observed between DP score and the structural accuracy (Fig. S1). The DP scores of most of the entries are above 0.7, with the large majority in the 0.85–0.95 range, indicative of excellent agreement between experimental data and structural results. These CASD-NMR-2013 data further suggest a more practical cutoff for DP scores of reliable models should be above ~0.75 (RMSDs to reference <~3 Å).

The expected number of NOEs that should be observed for a given structure ensemble presents a related measure correlating structural accuracy to experimental data. The so-called NOE completeness score can be calculated using the program Wattos, which is part of the CING suite. In practice, it is impossible to obtain all possible NOEs due to relaxation, peak overlap and alternating local conformations that can lead to conflicting assignments. In the NRG-CING database (Doreleijers et al. 2012b), the median NOE completeness is 44 %, and this represents a realistic goal for modern structure determination by NMR. Figure 4b shows the NOE completeness score for each of the targets and entries calculated from NOESY lists. For all the targets, the NOE completeness was well above the median database completeness consistent with the high quality of the target input data and the resulting structures. On average, the entries generated 64 % (range 31–87 %) of the expected number of restraints. With the exception of the algorithms using methods that rely exclusively on raw spectral data, i.e. UNIO and Ponderosa, all other entries produced assigned NOE peak lists that yielded a completeness well above the database median. In comparison to the targets, three tools (ARIA, CYANA and I-TASSER) performed better than the expert in assigning NOE peaks.

**Fig. 5:**
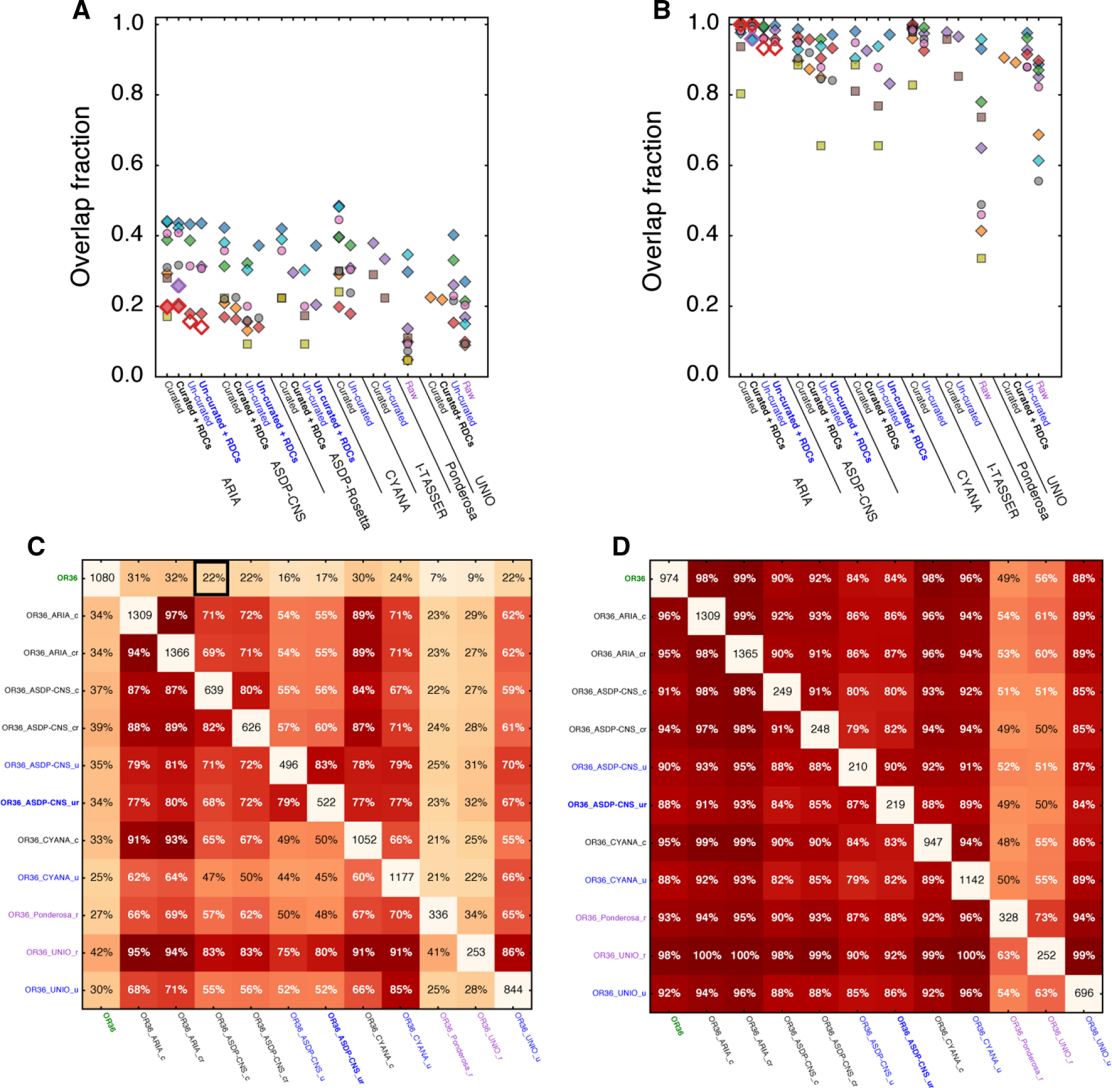
Overlap of long-range NOE restraints between the targets and the entries. Fraction of overlapping NOE restraints between the target and each entry determined on the basis of **a** pseudo-atom or **b** residue. *Symbols* and *labels* are explained in the legend for Fig. 1. **c**, **d** Heatmaps of the fractions of overlapping long-range NOE restraints between the OR36 target and entries, determined on the basis of pseudo-atom (**c**) or residue (**d**). The total number of long-range restraints present for each target/entry is shown on the diagonal. The off-diagonal values denote the percentage of restraints used in the entry indicated along the *row* that are also found in the entry indicated along the *column*. The *top row* shows the percentage of NOE’s used in the reference structure that were found in each entry, while the *left-most column* shows the percentage of NOE’s used by each entry that were found in the reference structure. For example, the entry in the *square* marked by the *black box* in (**c**) shows that 238 restraints (22 %) used in the OR36 target are also present in the OR36_ASDP-CNS_c entry

This high level of NOE completeness, combined with the high level of accuracy and the generally good quality of the structures generated, led us to hypothesize that there would be significant overlap between the restraints identified and used by the expert researcher and any one of the automated protocols as implemented in the different programs. To investigate this, we started from the list of restraints generated manually by the expert researcher or by each of the programs for each target. We removed the differences between the expert’s and the algorithms’ treatment of stereochemical assignment by treating all restraints as pseudo-atom restraints. This list was then curated to include only long-range restraints, i.e. those between atoms at least five residues apart, since these are the restraints that are known to carry the majority of the structural information (Nabuurs et al. 2003). The results of this analysis are shown in Fig. 5a. To our surprise they revealed that none of the automated methods identified more than 50 % of the restraints produced manually and some even obtained less than 10 %. As an example, Fig. 5c shows the restraint overlap between target OR36 and all entries (the majority of which were within 1.5 Å from the target structure). The aforementioned low degree of overlap between the manually derived, i.e. target, restraints is evident from the top row of this graph. The overlap between entries derived by the different programs is also highly variable, albeit that within a single program group the values are, as expected, consistently much higher.

Following this somewhat unexpected result, we decided to look at the overlap on a residue-to-residue basis rather than atom-to-atom, as it could be expected that the proximity of two residues rather than the exact NOE is the determining factor. Starting from the same curated pseudo-atom restraint lists generated above, we then generated a list containing only residue-to-residue restraint information. The results shown in Fig. 5b were much more consistent with our hypothesis, as for most of the targets the restraints obtained by the automated algorithms were now overlapping by more than 80 % with the manually derived restraints. A similar increase was obtained for the restraint overlap between the entries generated by the different programs, as illustrated for the OR36 target (Fig. 5d).

**Fig. 6:**
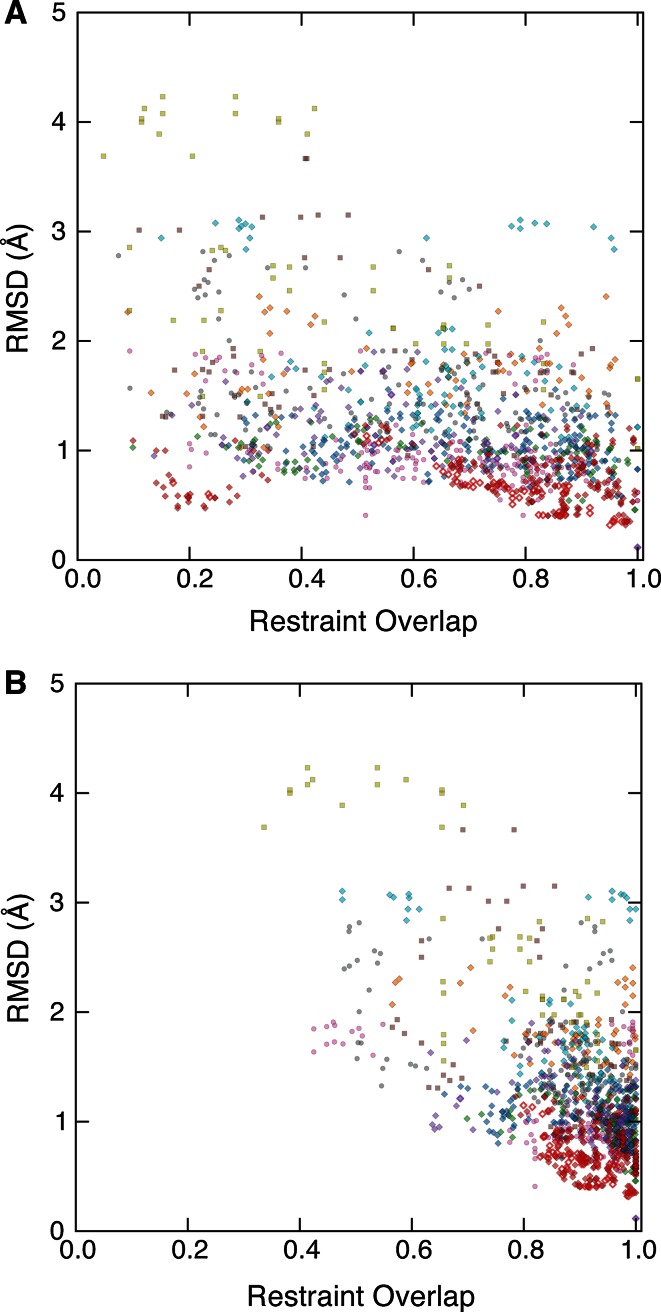
Correlation between entry pairwise RMSD and NOE restraint overlap. For every pair of entries for a given target, the all-by-all RMSD and NOE restraint overlap between those entries is shown. NOE restraint overlap are calculated on a **a** pseudo-atom or **b** residue basis. *Symbols* are explained in the legend for Fig. 1

Given the importance of NOE data in producing an accurate structure for many of the programs used in the CASD-NMR-2013 effort, we wanted to explore a possible correlation between the accuracy of the results, as expressed by RMSD (cf. Fig. 1) to the target, and the NOE-derived restraint overlap (Fig. 6). Although an extremely weak correlation can be inferred for some targets and methods (data not shown) no overall correlation patterns are supported by the data, neither on a pseudo-atom basis nor on a per-residue basis. Some clustering based on program group is also observed for some targets, however the clustering observed is not consistent across targets in either the pseudo-atom or per-residue based plots. We speculate that this unexpected lack of correlation can be explained by the information redundancy present in NOEs between atoms contained within a fixed covalent network.

It is clear from the figures in this paper that both RMSD values and validation scores are correlated for individual targets across different calculations; some targets tend to be either among the best or among the worst for all calculation protocols. The observation is semi-quantitative at best, given the variability of results across programs and program types, and the fact that not all targets were attempted (or resulted in converged structures) for all programs. It was not possible to determine any systematic variation with structure type: CASD-2013 included six α/β proteins and three all-α proteins, and both groups contained both ‘good’ and ‘bad’ targets. Two of the targets stood out for unrelated reasons, as discussed in the accompanying paper (Rosato et al. 2015). Producing converged entries from the un-curated peak lists for HR8254A and StT322 proved difficult, and entries for these targets were missing for a number of programs. These targets were both small (ca. 40 defined residues), were the only targets to use non-uniformly-sampled NMR data, had no RDC data, and had a high proportion of probable noise peaks in their un-curated peak lists. HR8254A gave consistently good validation scores, but had among the highest RMSD values for both accuracy and convergence. HR8254A is a three-helix protein with one very long helix extending outside a small core; clearly RMSD calculations will be quite sensitive to small variations in the inter-helical angle that, in the absence of RDC data, is difficult to determine precisely. StT322 is the only all-β protein in the set, and has a particularly large ill-defined tail. It gave consistently poor validation scores, and also gave high RMSD values for both convergence and accuracy.

## Discussion

The above results provide for a comprehensive evaluation of the performance of the currently available programs for automated protein structure generation from NMR data. The reference structures for all of the ten CASD-NMR-2013 targets are well converged (ensemble convergence 0.4–1.0 Å). The quality of these structures is higher than in the CASD-NMR-2010 round (ensemble convergence 0.4–1.7 Å) suggesting that either the quality of the input data has improved, most likely as a result of improved NMR hardware and acquisition schemes, or that there have been significant improvements in manual data analysis and structure calculation routines. Most likely, it resulted from a combination of both these factors.

Overall, the performance of the automated structure determination methods, in terms of accuracy and ensemble convergence, was excellent (median ensemble convergence 0.6 Å; median accuracy 1.14 Å). An accuracy threshold of 1.5 Å was imposed in this work to identify acceptable structures, which was achieved by 71 % of the entries. The less stringent 2.5 Å threshold imposed previously (Rosato et al. 2012) was achieved by 72 % of the entries in CASD-NMR-2010. Applying the same more relaxed criterion to the present CASD-NMR-2013 effort raises the success rate to 85 % of the entries. This improvement relative to CASD-NMR-2010 may partly be attributed to the quality of the input data as discussed above, but more likely also to advances in the structure generation engines, as also the overall quality indicators have improved. The success rate is even higher (78 % using the 1.5 Å cutoff) if the most challenging targets, HR8254A and StT322, are excluded. As for CASD-NMR-2010, the performance of the programs depended on the nature of the input data and once again the best results were obtained by methods using NOESY data, either as the primary input or to augment CS data.

The comparison between the entries obtained from either the use of curated NOESY peak lists *versus* un-curated peak lists revealed that for programs relying on NOESY data as the primary input the use of curated peak lists does not lead to significantly better structures. It appears that the iterative procedures implemented in these protocols are efficient at filtering the peaks for consistent information. For example, CYANA performs the so-called network-anchoring and restraint-combination methods to perform such peak filtering (Herrmann et al. 2002). However, programs such as Cheshire-YAPP that use NOESY data to augment the input CS data performed significantly better when using curated peak lists. The data also suggest that UNIO performs better with an un-curated peak list than with raw spectral data. The use of curated peak lists may however improve convergence. It is interesting to note that five out of ten ARIA calculations and three out of eight CYANA calculations with un-curated peaks failed to converge, where the equivalent calculations using curated peaks led to good quality structures. The non-converging seemed to correlate with the proportion of extraneous peaks in the un-curated peak lists (data not shown).

As for CASD-NMR-2010, three validation parameters were used to assess the geometric and packing quality of the CASD-NMR-2013 submissions: Ramachandran backbone angle distribution, side-chain angle distribution and the number of high-energy interatomic contacts (Fig. 2). As in the previous CASD-NMR-2010 round, these parameters varied over a wide range of Z-score (up to 15 standard deviations), and were overwhelmingly determined by the choice of structure calculation protocol. The three Rosetta-based protocols (Autonoe, CS-HM-Rosetta, and Web server Rosetta) and also the Rosetta-refined ASDP-Rosetta all did extremely well based on geometric criteria. This result is not surprising, given that these programs derive their backbone conformations and refinement parameters from databases of known good geometries. ARIA performed moderately well across all criteria, whereas ASDP-CNS, CYANA, Cheshire, and Ponderosa achieved acceptable but more mixed validation scores. Nevertheless, the relatively good validation scores for ARIA and ASDP-CNS confirm that water refinement in a realistic force field has a very positive effect on the geometry of the final conformer, also when performed in automation. Finally, UNIO and Cheshire-YAPP consistently received the lowest scores across all criteria, which is likely due to the lack of the aforementioned refinement procedures. Our investigations did not reveal any promising correlations between any of the geometric parameters and either accuracy or convergence.

Residue-specific ROG scores are indicators of local conformation and sensitive to errors in restraints. Within the set of entries, Rosetta/chemical-shift based methods show very good ROG scores (Fig. 3). Again, this is an expected result as some of the parameters that underpin the ROG score are also based upon comparison with fragments from structures contained within the PDB database. The results for Cheshire however, display an interesting phenomenon; whereas the chemical-shift-only entries display the expected good ROG scores, inclusion of the NOE peak lists does improve accuracy, albeit at the expense of much poorer ROG scores. It has been shown that for NOE/RDC-based structures consistently poor local conformation, as expressed by poor ROG scores, correlates with propensity for errors in the overall fold. It is notable that the entries for the StT322 target generally display among the worst ROG scores combined with the lowest accuracy scores (Fig. 1a). Generally however, the accuracy of the entries is high, suggesting that other factors may also depress the ROG scores. Proper refinement in a force-field that implements an explicit water-shell has been shown to substantially improve local conformation as well as the agreement with experimental restrains (Linge et al. 2003b; Spronk et al. 2002). Whereas some protocols, e.g. ARIA, ASDP and Autonoe, do implement such a refinement step as a standard procedure, for others like CYANA and UNIO this is generally not the case. As the accuracy of the latter protocols is similar to the accuracy of the former, it is likely that the observed differences in their ROG score patterns could be the result of the (lack of) final refinement, rather than of significant differences in the interpretation of the underlying data.

For the CASD-NMR-2013 entries we investigated the quality of the NOE input data and the accuracy of the structure generated by the different methods. For both the reference structures and the CASD-NMR-2013 entries the NOE completeness scores (Fig. 4b) were well-above the median in the CING database and the DP-scores (Fig. 4a) largely exceeded the lower cutoff of 0.7. Together, this indicates that all of the automated methods perform well with regard to assigning NOE restraints. We also determined the extent of NOE restraint overlap between different entries using either pseudo-atoms or residues as the basis for comparison (Fig. 5). When using pseudo-atoms as the basis for comparison, the restraint overlap was surprisingly low between the reference structure and the CASD-NMR-2013 entries but the degree of overlap increased significantly when determined at the residue level. This discrepancy between the extent of restraint overlap observed when using pseudo-atom to pseudo-atom restraints compared to residue to residue restraints could be explained by experimenter bias when manually generating restraint lists. For example, a human researcher, having identified NOE patterns consistent with an alpha helix, may invest more time and energy identifying all of the NOEs for the helix than the unbiased automated methods. Similarly, the human researcher may devote more attention to assigning as many NOE’s as possible in an under-restrained portion of an intermediate structure while the automated methods would be expected to spend equivalent amounts of effort on each region in the molecule. In support of this notion, we observed that at the pseudo-atom level the overlap between any two of the entries was higher when compared to the overlap between the target restraint list and any one of the entries. This is exemplified by the OR36 target (Fig. 5c, d). The results obtained for the pseudo-atom basis (Fig. 5c) show that, in general, overlap is greater between automated methods than between an automated method and the manual assignment. However, the overlap does not reach 100 % in either case, suggesting that differences cannot be entirely due to experimenter bias alone. It has been previously observed that multiple calculations starting from the same data may result in different restraint sets, with only a subset of restraints common to all calculations (Buchner and Güntert 2015). In contrast, there is little difference at the residue level in the extent of overlap between two automated algorithms or between an automated algorithm and the expert researcher (Fig. 5d). Notably, the methods based on raw spectral data have a lower overlap with all other methods, also on the per-residue basis. Given that they generated accurate structures for the majority of targets, this suggests that not all long-range contacts are equally important to define the correct protein fold. Finally, we found that there was no significant correlation between the extent of NOE restraint overlap and the accuracy of the structure. This result was surprising given the importance of NOE data in producing good quality structures. We suggest that this could be explained by the information redundancy present in NOEs between atoms contained within a fixed covalent network.

## Conclusions

The ten targets of CASD-NMR-2013 constitute a high-quality set of NMR structures, exemplifying the quality that can be attained by a skilled researcher using state-of-the-art techniques. Overall, the results from CASD-NMR-2013 demonstrate that automated structure determination protocols are capable of reliably producing structures of comparable accuracy and quality, at least for small, single domain proteins such as the ten targets tested. The most robust results appear to be obtained when NOESY peak lists are used either as the primary input data or to augment CS data, with limited need to manually refine such lists. Since no single method performed consistently better than the others for all ten targets it is advisable to use more than one program routinely and combine the results.

## Electronic supplementary material

Supplementary material 1 (PDF 163 kb)

Supplementary material 2 (PDF 5022 kb)
