# Supplementary material for: Analysis of the structural quality of the CASD-NMR 2013 entries
Source: J Biomol NMR. 2015 Jun 3;62(4):527–40. doi: 10.1007/s10858-015-9949-0 (PMC4569653; doi:10.1007/s10858-015-9949-0)
Supplement: Supplementary file 1 — Supplementary material 1 (PDF 163 kb) [file 10858_2015_9949_MOESM1_ESM.pdf]

Supplementary Table 1.

EntryID	Target	Program Type	Entry	Invalid	Conformers	Accuracy	Convergence	ROG	NOE completeness	DP Score	WI packing quality	MP Clash Z-score	MP Clash score (pdb style)	WI Ramachandran appearance	MP % Ramachandran disallowed	PC_NMR ϕ/ψ Z-score	WI χ Z-	PC_NMR dihedral Z-score	Intra-NOEs	Seq-NOEs	Med-NOEs	Long-NOEs	Ambig-NOEs	Queen Information	NOE violations > 0.1 Å	NOE violations > 0.3 Å	NOE violations > 0.5 Å	dihedrals	dihedral violations > 1°	dihedral violations > 3°	dihedral violations > 5°	RDCs
HR2876B	HR2876B		HR2876B		20		0.62	green	73.2	0.93	2.10	0.02	9.06	-1.93	0	-0.04	-0.44	-0.30	699	628	519	1147	0	66.278	119	11	0	112	81	38	15	0
255	HR2876B	ARIA	HR2876B_ARIA_c		10	0.72	0.36	green	75.2	0.93	2.09	-0.74	12.36	-1.55	2.1	-0.35	-0.45	-0.53	619	1333	451	942	580	45.138	208	112	53	172	173	91	59	0
256	HR2876B	ARIA	HR2876B_ARIA_cr		10	0.78	0.53	green	75.5	0.93	2.00	-1.32	16.63	-2.24	3.4	-0.98	-0.67	-1.06	627	1340	447	946	609	75.970	315	202	143	172	272	170	138	121
240	HR2876B	ARIA	HR2876B_ARIA_u		10	0.91	0.58	green	74.4	0.90	2.42	-3.56	27.49	-2.23	1.6	-0.98	-2.37	-1.54	1041	1224	1024	2180	1447	66.538	7755	6749	5793	172	267	175	107	0
241	HR2876B	ARIA	HR2876B_ARIA_ur		10	1.05	0.70	green	74.5	0.91	1.72	-4.18	31.03	-3.06	2.4	-1.77	-2.14	-2.37	1057	1201	1067	2132	1461	80.438	7473	6483	5580	172	497	345	257	121
313	HR2876B	ASDP-CNS	HR2876B_ASDP-CNS_c		20	0.97	0.54	green	66.4	0.92	2.05	-1.70	21.32	-1.82	0.1	-0.59	-1.51	-1.01	493	441	381	759	0	87.979	85	0	0	164	362	116	64	0
278	HR2876B	ASDP-CNS	HR2876B_ASDP-CNS_ur		15	1.00	0.82	green	67.1	0.91	1.35	-0.92	15.57	-2.41	0.1	-0.55	-2.95	-1.42	569	429	440	809	0	75.689	189	9	0	164	422	210	118	120
314	HR2876B	ASDP-Rosetta	HR2876B_ASDP-Rosetta_c		20	0.76	0.38	green	66.8	0.93	2.36	0.66	4.96	-0.03	0	0.24	5.00	1.12	500	440	387	751	0	94.678	104	36	9	164	198	167	142	0
279	HR2876B	ASDP-Rosetta	HR2876B_ASDP-Rosetta_ur		20	0.88	0.53	green	66.2	0.92	2.04	-0.22	10.32	-0.42	0	0.28	2.86	0.83	569	429	440	809	0	75.689	466	309	221	164	183	118	88	120
244	HR2876B	autonoe-Rosetta-alpha	HR2876B_autonoe-Rosetta-alpha_ur		10	1.50	1.17	green			1.77	0.99	2.78	-1.36	0.8	-0.59	5.07	0.41	0	0	0	0	0		0	0	0	0	215	183	143	0
325	HR2876B	Cheshire	HR2876B_Cheshire_s		10	3.06	3.19	green			0.37		8.21	-3.47			-1.09		0	0	0	0	0		0	0	0	0	360	323	284	0
326	HR2876B	Cheshire-YAPP	HR2876B_Cheshire-YAPP_c		50	0.80	0.46	orange		0.93	0.60		19.36	-5.16			-6.86		0	0	0	0	0		0	0	0	0	2576	2291	1979	0
262	HR2876B	Cheshire-YAPP	HR2876B_Cheshire-YAPP_u		100	1.00	0.48	orange		0.91	1.52	-10.04	30.71	-3.21	1.6	-1.57	-7.20	-4.91	0	0	0	0	0		0	0	0	0	1585	991	675	0
283	HR2876B	CS-HM-Rosetta	HR2876B_CS-HM-Rosetta_sr		1	2.30		orange			-1.72			-2.19			6.46		0	0	0	0	0		0	0	0	0	27	25	20	0
259	HR2876B	CYANA	HR2876B_CYANA_c		20	0.71	0.38	green	76.1	0.93	1.79	0.67	5.13	-2.57	0.1	-1.02	-4.08	-2.31	675	592	525	1184	0	90.712	22	0	0	0	386	283	210	0
227	HR2876B	Ponderosa	HR2876B_Ponderosa_r		20	1.29	0.11	orange		0.89	0.52	-2.24	23.86	-0.55	1	-0.35	-6.03	-2.42	577	482	380	795	0	67.914	64	0	0	316	513	417	366	0
239	HR2876B	UNIO	HR2876B_UNIO_r		20	1.05	0.58	orange	54.8	0.89	0.27	-5.09	36.05	-4.78	0.4	-2.52	-6.63	-5.14	462	435	265	579	0	66.952	238	51	27	274	1092	956	851	0
238	HR2876B	UNIO	HR2876B_UNIO_u		20	1.03	0.45	red	65.5	0.87	0.84	-12.88	75.19	-5.56	3.1	-3.62	-6.74	-6.39	795	405	617	1228	0	88.163	521	134	82	274	1279	1091	958	0
HR2876C	HR2876C		HR2876C		20		0.53	green	68.0	0.89	2.41	-0.63	14.75	-1.70	0	0.12	-0.39	0.24	744	648	739	907	0	54.959	105	2	0	120	104	32	7	0
303	HR2876C	ARIA	HR2876C_ARIA_cr		15	1.05	0.12	green	62.5	0.90	2.39	-1.23	20.13	-1.32	2.8	-0.39	-0.70	-0.47	431	1008	451	500	325	54.387	94	19	12	140	251	175	152	95
304	HR2876C	ARIA	HR2876C_ARIA_cr(10-97)		15	1.05	0.12	green	62.5	0.90	2.39	-1.50	20.13	-1.32	2.8	-0.39	-0.70	-0.47	431	1008	451	500	325	54.387	94	19	12	140	240	164	142	95
296	HR2876C	ARIA	HR2876C_ARIA_ur		15	0.81	0.42	green	61.4	0.86	3.18	-2.23	26.59	-0.18	0.1	-0.04	-0.42	-0.18	664	936	688	795	440	72.045	1434	478	169	140	280	159	141	95
307	HR2876C	ASDP-Rosetta	HR2876C_ASDP-Rosetta_cr		20	0.76	0.51	green	62.2	0.89	2.91	-0.02	8.87	0.93	0	0.79	3.91	1.77	529	425	515	506	0	82.340	1311	426	111	158	200	121	85	0
301	HR2876C	ASDP-Rosetta	HR2876C_ASDP-Rosetta_ur		20	1.41	0.65	green	58.2	0.83	1.93	-1.20	15.18	-0.07	0	0.67	2.08	1.12	569	393	507	443	0	80.670	1299	557	229	158	334	251	184	0
288	HR2876C	autonoe-Rosetta-alpha	HR2876C_autonoe-Rosetta-alpha_ur		10	1.16	0.79	green		0.85	3.18	0.92	3.63	0.45	0	0.79	6.20	2.19	0	0	0	0	0		0	0	0	0	138	98	76	0
310	HR2876C	BE-metadynamics	HR2876C_BE-metadynamics_s		28	1.68	1.66	green		0.77	0.70	1.13	2.35	-3.97	0.4	-1.06	-4.91	-2.07	0	0	0	0	0		0	0	0	0	1036	859	725	0
309	HR2876C	Cheshire	HR2876C_Cheshire_s		10	1.84	1.32	green			2.05	-1.70	19.03	-2.14	0.6	-0.04	-0.68	0.06	0	0	0	0	0		0	0	0	0	324	291	254	0
308	HR2876C	Cheshire-YAPP	HR2876C_Cheshire-YAPP_c		50	1.05	0.56	red		0.90	1.68	-5.08	32.06	-5.05	1.3	-2.36	-7.62	-5.03	0	0	0	0	0		0	0	0	0	2389	2040	1773	0
300	HR2876C	Cheshire-YAPP	HR2876C_Cheshire-YAPP_u		50	2.07	1.14	red		0.85	0.01	-10.58	58.46	-7.18	4.9	-4.48	-7.74	-7.69	0	0	0	0	0		0	0	0	0	3896	3627	3396	0
291	HR2876C	CS-HM-DP-Rosetta	HR2876C_CS-HM-DP-Rosetta_ur		11	1.40	0.86	green		0.81	1.93	0.89	3.85	-1.68	0.7	-0.28	6.08	0.83	0	0	0	0	0		0	0	0	0	290	248	206	0
285	HR2876C	CS-HM-Rosetta	HR2876C_CS-HM-Rosetta_sr		11	1.88	0.42	green			1.99	0.99	3.30	-0.15	0	0.12	6.31	1.24	0	0	0	0	0		0	0	0	0	245	197	171	0
286	HR2876C	CS-Rosetta	HR2876C_CS-Rosetta_s		10	0.96	0.82	green			3.07	0.19	6.57	0.01	0	0.75	5.75	1.89	0	0	0	0	0		0	0	0	0	125	105	78	0
287	HR2876C	CS-Rosetta3	HR2876C_CS-Rosetta3_s		6	1.00	0.85	green			2.70	0.55	5.91	0.01	0	0.59	6.62	1.83	0	0	0	0	0		0	0	0	0	95	74	64	0
302	HR2876C	CYANA	HR2876C_CYANA_c		20	0.84	0.34	green	71.0	0.89	1.83	-0.20	12.37	-2.52	0	-0.16	-5.42	-1.60	739	607	735	940	0	73.930	64	0	0	0	466	338	274	0
297	HR2876C	CYANA	HR2876C_CYANA_u		20	0.97	0.30	orange	66.8	0.87	1.49	-2.03	21.89	-4.40	2.6	-1.65	-6.15	-3.61	724	615	690	879	0	74.135	111	0	0	0	750	612	502	0
305	HR2876C	I-TASSER	HR2876C_I-TASSER_c		1	1.32		green	86.2	0.82	-1.14	-1.19	18.17	-0.50	0	0.04	-3.21	-1.30	1781	2253	1792	2011	275	89.898	216	75	28	138	32	24	16	0
298	HR2876C	I-TASSER	HR2876C_I-TASSER_u		1	1.11		green	86.3	0.76	-1.66	-0.03	10.38	-2.06	2.3	-0.94	-2.24	-2.07	1867	1660	1970	2259	181	95.111	246	121	71	138	57	42	37	0
284	HR2876C	Ponderosa	HR2876C_Ponderosa_r		20	0.95	0.17	orange		0.83	1.64	-2.66	27.68	-1.14	0	-0.04	-7.22	-2.72	526	428	419	326	0	31.966	3829	2463	1688	307	863	733	621	0
293	HR2876C	UNIO	HR2876C_UNIO_r		20	1.32	0.63	orange	48.0	0.86	1.03	-3.52	34.99	-5.13	0.1	-1.81	-6.94	-4.67	404	406	314	348	0	55.568	332	167	108	259	1218	1017	907	0
294	HR2876C	UNIO	HR2876C_UNIO_u		20	1.12	0.50	red	57.7	0.86	0.84	-7.89	64.62	-6.04	0.3	-2.91	-7.24	-5.62	648	395	624	678	0	69.220	424	166	122	258	1286	1054	937	0
HR5460A	HR5460A		HR5460A		20		0.60	green	67.8	0.87	2.43	-1.36	18.85	-1.63	0	1.49	-1.71	0.83	1063	1139	1422	1349	0	47.608	408	11	0	220	61	7	1	0
176	HR5460A	ARIA	HR5460A_ARIA_c		10	1.56	0.67	green	77.1	0.87	2.64	-2.76	25.98	-1.83	0.6	1.26	-1.58	0.65	1306	2657	1600	1547	3120	30.707	3511	2057	1241	266	468	288	189	0
177	HR5460A	ARIA	HR5460A_ARIA_cr		10	1.24	0.68	green	77.4	0.86	2.80	-2.72	25.93	-1.28	0.5	1.26	-1.58	0.59	1307	2653	1588	1539	3105	64.508	3528	2139	1312	266	397	241	161	83
331	HR5460A	ARIA	HR5460A_ARIA_u	TRUE	10	11.40	1.60	red	51.4	0.52	-2.26		235.71	-7.47			-7.48		1398	1750	1512	1368	1615	65.178	5903	2877	1566	266	2590	2183	1833	0
173	HR5460A	ASDP-CNS	HR5460A_ASDP-CNS_c		20	1.70	0.90	green	64.3	0.87	2.29	-0.94	15.14	-2.12	0.1	1.30	-2.45	0.24	876	774	1006	770	0	76.358	965	63	5	272	955	612	433	0
174	HR5460A	ASDP-CNS	HR5460A_ASDP-CNS_cr		20	1.52	0.80	green	62.1	0.86	2.47	-1.61	17.20	-2.08	0	1.38	-2.31	0.35	858	772	1041	773	0	78.956	971	60	3	272	980	658	465	83
164	HR5460A	ASDP-CNS	HR5460A_ASDP-CNS_u		20	1.53	1.15	green	57.0	0.84	2.01	-1.00	15.99	-2.27	0	1.34	-3.22	-0.18	831	733	920	612	0	39.015	1030	85	14	274	1078	687	433	0
165	HR5460A	autonoe-Rosetta-alpha	HR5460A_autonoe-Rosetta-alpha_ur		10	3.46	2.56	green			2.85	0.86	4.17	-0.02	0	2.01	6.90	2.78	0	0	0	0	0		0	0	0	0	259	181	129	0
321	HR5460A	Cheshire	HR5460A_Cheshire_s		1	3.52		green		0.61	-0.01		5.66	-0.96			2.50		0	0	0	0	0		0	0	0	0	32	20	17	0
323	HR5460A	Cheshire-YAPP	HR5460A_Cheshire-YAPP_c		10	1.11	0.69	orange		0.87	0.61		37.47	-6.28			-7.32		0	0	0	0	0		0	0	0	0	1072	897	750	0
322	HR5460A	Cheshire-YAPP	HR5460A_Cheshire-YAPP_u	TRUE	35	5.78	4.85	red		0.75	-2.17		22.63	-8.31			-8.45		0	0	0	0	0		0	0	0	0	6573	6234	5919	0
138	HR5460A	CS-HM-Rosetta	HR5460A_CS-HM-Rosetta_sr		10	1.70	1.33	green			3.58	0.62	5.48	1.00	0	2.32	6.95	2.96	0	0	0	0	0		0	0	0	0	201	145	103	0
168	HR5460A	CS-Rosetta	HR5460A_CS-Rosetta_s		5	3.12	2.87	green		0.75	3.28	0.02	9.32	0.82	0	2.20	6.88	2.84	0	0	0	0	0		0	0	0	0	111	63	38	0
171	HR5460A	CYANA	HR5460A_CYANA_c		20	1.03	0.44	green	67.5	0.87	1.96	-0.03	10.13	-2.78	1.4	0.83	-5.52	-1.66	1100	1149	1402	1437	0	70.903	124	0	0	0	842	582	407	0
175	HR5460A	Ponderosa	HR5460A_Ponderosa_r		20	9.33	0.93	orange		0.69	1.81	-5.54	41.76	-1.06	0	1.57	-7.57	-2.78	865	747	786	288	0	27.522	5890	4473	3569	531	1134	835	639	0
172	HR5460A	UNIO	HR5460A_UNIO_c		20	1.22	0.67	orange	61.6	0.86	0.72	-0.48	12.98	-4.45	1.3	-0.20	-6.54	-3.25	864	975	1109	926	0	72.621	874	381	316	419	1597	1237	994	0
191	HR5460A	UNIO	HR5460A_UNIO_cr		20	1.31	0.61	orange	60.3	0.87	1.28	-0.57	13.09	-3.90	1	0.04	-6.90	-3.25	888	992	1125	916	0	70.561	911	416	298	419	1480	1149	931	0
162	HR5460A	UNIO	HR5460A_UNIO_r		20	2.26	1.40	orange	36.6	0.83	0.09	-2.70	27.52	-6.45	0	-1.61	-7.28	-4.61	475	674	375	376	0	44.366	405	193	150	417	2284	1946	1688	0
HR6430A	HR6430A		HR6430A		20		0.52	green	79.7	0.94	2.66	-0.69	14.33	-1.55	0	-0.31	-0.98	-0.30	658	570	479	1211	0	41.613	174	3	0	80	44	24	18	0
166	HR6430A	ARIA	HR6430A_ARIA_c		10	0.80	0.46	green	82.2	0.94	2.97	-1.19	15.89	-1.04	1.2	-0.79	-0.90	-0.77	751	1310	552	1331	1290	38.006	817	451	247	158	311	212	132	0
144	HR6430A	ARIA	HR6430A_ARIA_u		10	0.82	0.43	green	81.1	0.94	3.02	-1.41	16.87	-0.96	1.2	-0.63	-0.96	-0.65	749	1309	556	1336	1290	36.959	844	498	255	158	316	220	139	0
167	HR6430A	ASDP-CNS	HR6430A_ASDP-CNS_c		20	1.08	0.81	green	71.9	0.92	2.59	-0.72	14.49	-1.55	0.1	-0.75	-2.56	-1.42	491	421	338	763	0	78.591	291	7	0	152	610	415	314	0
148	HR6430A	ASDP-CNS	HR6430A_ASDP-CNS_u		20	0.95	0.77	green	69.6	0.91	2.33	-0.72	14.67	-1.60	0.1	-0.87	-2.64	-1.66	503	426	352	807	0	82.795	353	28	0	155	640	424	301	0
147	HR6430A	autonoe-Rosetta-alpha	HR6430A_autonoe-Rosetta-alpha_ur		10	1.79	1.82	green		0.84	2.36	0.72	3.25	-0.47	0	-0.12	5.35	0.95	0	0	0	0	0		0	0	0	0	216	184	156	0
145	HR6430A	Cheshire	HR6430A_Cheshire_s		2	1.19	0.00	red			-1.12	-1.75	32.10	-3.46	5.7	-3.42	-5.20	-4.55	0	0	0	0	0		0	0	0	0	86	80	56	0
169	HR6430A	Cheshire-YAPP	HR6430A_Cheshire-YAPP_c		100	0.77	0.40	orange		0.94	1.71	-3.53	13.43	-4.77	1.2	-2.60	-6.51	-4.44	0	0	0	0	0		0	0	0	0	4308	3825	3372	0
146	HR6430A	Cheshire-YAPP	HR6430A_Cheshire-YAPP_u		100	1.03	0.75	red		0.94	1.33	-4.60	13.38	-5.73	1.6	-3.19	-6.43	-5.38	0	0	0	0	0		0	0	0	0	5046	4529	4090	0
150	HR6430A	CS-DP-Rosetta	HR6430A_CS-DP-Rosetta_u		5	2.02	2.30	green		0.80	1.78	-0.66	10.45	0.25	0	0.00	4.14	0.71	0	0	0	0	0		0	0	0	0	94	74	59	0
149	HR6430A	CS-HM-Rosetta	HR6430A_CS-HM-Rosetta_sr		10	1.21	0.59	green		0.89	3.01	0.66	4.92	0.76	0	0.08	5.17	1.18	0	0	0	0	0		0	0	0	0	185	153	130	0
152	HR6430A	CYANA	HR6430A_CYANA_c		20	0.84	0.36	green	79.2	0.93	1.66	0.89	4.16	-1.71	1.2	-1.22	-4.72	-2.31	656	582	470	1204	0	79.969	10	0	0	0	417	337	284	0
134	HR6430A	CYANA	HR6430A_CYANA_u		20	0.90	0.34	green	76.0	0.93	1.80	0.72	5.30	-2.23	0.1	-1.30	-5.57	-2.54	684	572	507	1220	0	86.010	19	0	0	0	446	364	301	0
163	HR6430A	Ponderosa	HR6430A_Ponderosa_r		20	1.03	0.05	orange	41.1	0.85	0.67	-3.73	34.94	0.11	0.1	-0.31	-7.21	-3.13	514	441	294	497	0	49.395	5484	3685	2485	100	1141	676	445	0
141	HR6430A	UNIO	HR6430A_UNIO_r		20	1.13	0.74	green	59.8	0.92	1.04	0.84	4.39	-3.58	0.7	-1.65	-5.11	-3.25	420	365	221	578	0	60.817	157	65	56	253	665	547	479	0
142	HR6430A	UNIO	HR6430A_UNIO_u		20	0.91	0.73	orange	72.3	0.94	1.34	0.48	6.70	-3.60	0.3	-1.49	-5.62	-3.13	610	523	432	1006	0	78.679	194	33	13	255	587	501	436	0

EntryID	Target	Program Type	Entry	Invalid	Conformers	Accuracy	Convergence	ROG	NOE completeness	DP Score	WI packing quality	MP Clash Z-score	MP Clash score (pdb style)	WI Ramachandran appearance	MP % Ramachandran disallowed	PC_NMR ϕ/ψ Z-score	WI χ Z-	PC_NMR dihedral Z-score	Intra-NOEs	Seq-NOEs	Med-NOEs	Long-NOEs	Ambig-NOEs	Queen Information	NOE violations > 0.1 Å	NOE violations > 0.3 Å	NOE violations > 0.5 Å	dihedrals	dihedral violations > 1°	dihedral violations > 3°	dihedral violations > 5°	RDCs
HR6470A	HR6470A		HR6470A		20		0.40	green	68.4	0.91	2.32	0.76	12.87	-0.09	0	1.57	1.62	2.66	368	429	462	313	0	31.906	66	2	0	140	28	10	0	73
154	HR6470A	ARIA	HR6470A_ARIA_c		10	0.57	0.38	green	81.7	0.91	2.76	0.16	7.95	0.26	0	1.97	1.17	1.77	414	1011	448	314	633	21.942	281	192	126	92	87	55	36	0
156	HR6470A	ARIA	HR6470A_ARIA_c(10-59)		10	0.58	0.30	green	80.9	0.88	3.04	0.01	10.11	0.53	0	2.08	0.42	1.66	397	878	443	310	518	34.692	312	217	142	92	83	56	26	0
155	HR6470A	ARIA	HR6470A_ARIA_cr		9	0.57	0.32	green	80.1	0.91	2.97	-0.10	10.78	1.09	0	2.12	1.59	2.19	430	1009	442	304	633	30.763	255	185	113	92	58	42	26	73
157	HR6470A	ARIA	HR6470A_ARIA_cr(10-59)		10	0.60	0.37	green	79.9	0.88	2.87	-0.07	10.78	0.36	0	2.01	0.76	1.77	386	874	446	316	509	24.788	320	221	162	92	66	47	30	73
129	HR6470A	ARIA	HR6470A_ARIA_u		10	0.49	0.44	green	77.2	0.91	2.88	-0.48	12.94	0.81	0.2	1.81	1.12	1.71	444	1002	461	284	667	38.921	395	288	182	92	60	38	26	0
128	HR6470A	ARIA	HR6470A_ARIA_u(10-59)		10	0.58	0.36	green	76.5	0.88	2.88	-0.47	13.61	0.72	0	1.85	1.07	1.42	404	878	458	260	508	34.670	477	335	232	92	91	54	35	0
127	HR6470A	ARIA	HR6470A_ARIA_ur		10	0.47	0.36	green	76.5	0.90	2.98	-0.30	11.19	0.60	0	2.12	1.48	1.89	443	1004	456	281	650	30.880	393	261	184	92	120	74	54	73
126	HR6470A	ARIA	HR6470A_ARIA_ur(10-59)		10	0.70	0.53	green	78.2	0.86	2.78	-0.23	11.46	0.53	0	2.08	0.48	1.71	409	880	466	261	555	23.567	542	407	280	92	90	61	42	73
158	HR6470A	ASDP-CNS	HR6470A_ASDP-CNS_c		20	0.73	0.72	green	67.2	0.90	2.13	0.13	11.12	-1.31	0	1.73	-1.39	0.83	288	320	315	156	0	62.887	145	5	1	106	358	219	128	0
159	HR6470A	ASDP-CNS	HR6470A_ASDP-CNS_cr		20	0.78	0.80	green	58.8	0.90	2.19	-0.13	13.48	-1.45	0	1.69	-1.50	0.83	231	162	309	155	0	36.233	72	5	0	106	335	200	120	73
136	HR6470A	ASDP-CNS	HR6470A_ASDP-CNS_u		20	1.00	0.51	green	58.7	0.85	2.77	-1.11	23.65	-0.14	0	0.83	-2.40	0.06	297	322	329	144	0	63.040	658	515	425	106	240	97	42	0
135	HR6470A	ASDP-CNS	HR6470A_ASDP-CNS_ur		20	1.00	0.51	green	60.1	0.85	2.77	-1.11	23.65	-0.14	0	0.83	-2.40	0.06	302	323	328	140	0	62.120	73	3	3	106	240	97	42	0
185	HR6470A	autonoe-Rosetta-alpha	HR6470A_autonoe-Rosetta-alpha_u(11-58)		10	0.78	0.83	green			3.47	-0.47	4.99	1.28	0	1.85	7.96	1.42	0	0	0	0	0		0	0	0	0	61	39	25	0
186	HR6470A	autonoe-Rosetta-alpha	HR6470A_autonoe-Rosetta-alpha_ur		10	0.83	0.77	green		0.88	3.01	0.76	6.75	0.63	0	1.57	7.29	2.66	0	0	0	0	0		0	0	0	0	98	72	48	0
131	HR6470A	Cheshire	HR6470A_Cheshire_s		1	0.81		green			-0.18			1.02			0.29		0	0	0	0	0		0	0	0	0	5	3	0	0
153	HR6470A	Cheshire-YAPP	HR6470A_Cheshire-YAPP_c		20	0.57	0.38	orange		0.89	1.81	-0.32	15.61	-4.35	0	-0.08	-6.16	-3.08	0	0	0	0	0		0	0	0	0	527	399	302	0
132	HR6470A	Cheshire-YAPP	HR6470A_Cheshire-YAPP_u		50	0.64	0.34	red		0.87	1.50	0.05	12.11	-4.77	0.4	-0.39	-6.28	-3.49	0	0	0	0	0		0	0	0	0	1538	1203	908	0
143	HR6470A	CS-HM-Rosetta	HR6470A_CS-HM-Rosetta_sr		10	0.54	0.32	green		0.89	3.20	1.01	4.58	1.38	0	2.36	7.00	3.13	0	0	0	0	0		0	0	0	0	60	36	26	0
121	HR6470A	CS-Rosetta	HR6470A_CS-Rosetta_s		5	0.62	0.46	green		0.84	2.93	0.06	8.65	0.37	0	2.12	7.37	3.02	0	0	0	0	0		0	0	0	0	38	22	17	0
137	HR6470A	CYANA	HR6470A_CYANA_c		20	0.53	0.42	green	73.0	0.91	2.30	1.27	2.16	-0.85	0	1.65	-2.53	0.59	357	438	428	327	0	42.061	2	0	0	0	220	146	90	0
133	HR6470A	CYANA	HR6470A_CYANA_u		20	0.59	0.46	green	72.2	0.90	1.92	1.07	3.78	-1.54	0	1.61	-2.91	0.53	385	461	397	262	0	36.170	7	0	0	0	242	152	92	0
125	HR6470A	Ponderosa	HR6470A_Ponderosa_r		20	0.65	0.07	green		0.83	2.28	-0.81	20.95	1.63	0	1.22	-6.08	-1.83	399	401	361	258	0	53.100	2	0	0	256	421	333	266	0
122	HR6470A	UNIO	HR6470A_UNIO_r		20	1.09	0.61	green	52.8	0.86	0.75	1.25	2.36	-2.55	2.1	0.90	-4.77	-1.42	206	291	179	135	0	28.179	23	7	4	188	519	416	360	0
123	HR6470A	UNIO	HR6470A_UNIO_u		20	0.66	0.51	orange	65.2	0.90	2.30	1.03	4.05	-1.32	0	1.42	-4.65	-0.83	334	417	344	204	0	37.227	72	17	14	185	323	238	157	0
HR8254A	HR8254A		HR8254A		20		0.72	green	59.2	0.83	2.79	-0.88	15.61	-2.26	0.1	1.53	-1.30	1.01	387	408	444	300	0	29.160	999	811	746	125	64	2	0	0
273	HR8254A	ARIA	HR8254A_ARIA_c		10	1.50	0.52	green	66.0	0.83	4.23	-1.50	22.61	0.90	0	2.48	1.10	2.66	362	865	396	242	325	48.112	377	135	66	130	80	23	16	0
335	HR8254A	ARIA	HR8254A_ARIA_u	TRUE	10	10.71	0.70	orange	57.2	0.42	-1.04		68.05	-5.81			-3.20		648	852	584	669	497	61.400	1988	1333	857	130	1100	729	493	0
275	HR8254A	ASDP-Rosetta	HR8254A_ASDP-Rosetta_c		20	1.74	1.08	green	55.8	0.84	4.17	-0.03	6.79	0.81	1.8	2.44	6.51	3.55	315	336	414	149	0	69.961	959	229	48	108	521	470	425	0
274	HR8254A	ASDP-Rosetta	HR8254A_ASDP-Rosetta_u		20	1.73	1.32	green	54.0	0.81	3.23	-0.08	9.55	0.75	0.6	1.97	4.58	2.72	305	316	403	128	0	70.915	1068	350	112	110	590	506	449	0
265	HR8254A	autonoe-Rosetta-alpha	HR8254A_autonoe-Rosetta-alpha_c		10	2.68	0.99	green		0.81	3.05	1.35	1.17	0.37	0	2.05	7.50	3.25	0	0	0	0	0		0	0	0	0	94	71	59	0
264	HR8254A	autonoe-Rosetta-alpha	HR8254A_autonoe-Rosetta-alpha_u		10	3.91	0.97	green		0.73	3.84	1.01	3.29	1.30	0	2.24	6.89	3.19	0	0	0	0	0		0	0	0	0	145	116	102	0
329	HR8254A	Cheshire	HR8254A_Cheshire_s		1	2.77		green			0.22			-1.75			1.90		0	0	0	0	0		0	0	0	0	35	30	23	0
270	HR8254A	CS-HM-Rosetta	HR8254A_CS-HM-Rosetta_s		11	1.38	0.48	green		0.81	4.21	1.40	0.97	1.49	0	2.60	8.49	3.78	0	0	0	0	0		0	0	0	0	78	59	47	0
269	HR8254A	CS-Rosetta	HR8254A_CS-Rosetta_s		5	1.52	1.31	green		0.79	4.18	0.93	3.82	1.80	0	2.71	9.25	4.02	0	0	0	0	0		0	0	0	0	36	25	14	0
299	HR8254A	CYANA	HR8254A_CYANA_c		20	1.04	0.80	green	61.3	0.82	2.44	0.72	5.89	-2.18	0	1.26	-3.56	0.06	368	403	444	295	0	56.637	20	0	0	0	375	279	227	0
271	HR8254A	I-TASSER	HR8254A_I-TASSER_c		10	1.31	0.28	green	86.6	0.77	3.84	-1.04	19.32	3.81	0	3.11	0.02	2.37	1241	1562	1630	854	65	56.519	863	392	229	130	64	27	7	0
272	HR8254A	I-TASSER	HR8254A_I-TASSER_u		10	1.31	0.25	green	80.0	0.74	3.22	-2.75	32.27	2.62	0	2.56	-0.80	1.71	1616	1434	1925	534	92	65.600	1486	806	552	130	128	90	43	0
268	HR8254A	Ponderosa	HR8254A_Ponderosa_r		20	3.01	0.02	green	38.9	0.75	2.59	-1.76	24.89	-0.62	0	2.36	-6.10	-0.18	398	451	366	211	0	41.794	3493	2768	2239	95	845	421	141	0
263	HR8254A	UNIO	HR8254A_UNIO_u		20	1.45	0.95	green	49.2	0.81	2.81	-4.24	38.69	-2.52	0.1	1.49	-2.69	0.53	267	341	278	167	0	38.784	116	0	0	140	579	436	359	0
OR135	OR135		OR135		20		0.64	green	71.9	0.90	4.06	-0.81	14.78	-1.70	0	0.31	-0.29	0.41	643	682	625	967	0	56.263	102	7	1	108	122	32	3	0
215	OR135	ARIA	OR135_ARIA_c		10	0.74	0.34	green	77.4	0.90	5.26	-1.03	15.16	0.80	0	0.79	0.90	0.95	560	1113	532	784	767	65.349	201	113	71	128	98	63	41	0
214	OR135	ARIA	OR135_ARIA_cr		10	0.84	0.49	green	76.8	0.90	4.01	-0.91	14.57	-2.20	0	-0.35	-0.13	-0.12	565	1133	531	788	770	52.826	226	152	92	128	274	184	132	104
196	OR135	ARIA	OR135_ARIA_u		10	0.81	0.45	green	68.4	0.89	4.87	-1.19	16.61	0.09	0	0.43	-0.48	0.18	457	930	433	641	666	66.264	482	283	173	128	147	92	72	0
199	OR135	ARIA	OR135_ARIA_ur		10	1.00	0.60	green	69.6	0.89	3.89	-1.76	18.65	-2.25	0.2	-0.28	-0.81	-0.41	462	924	408	652	636	53.221	509	341	226	128	360	240	157	104
312	OR135	ASDP-CNS	OR135_ASDP-CNS_c		20	0.88	0.46	green	64.9	0.89	4.94	-2.34	26.02	-0.86	0	-0.04	-1.10	-0.59	445	435	401	590	0	83.467	133	4	0	138	276	113	59	0
207	OR135	ASDP-CNS	OR135_ASDP-CNS_u		20	1.06	0.56	green	54.6	0.86	4.79	-2.14	24.57	-0.75	0	0.00	-2.07	-0.95	334	379	273	388	0	39.594	46	0	0	136	308	145	103	0
311	OR135	ASDP-Rosetta	OR135_ASDP-Rosetta_c		20	0.83	0.14	green	65.8	0.89	5.64	0.88	4.34	2.51	0	0.47	5.54	1.36	445	435	401	590	0	83.467	366	113	44	138	56	47	45	0
208	OR135	ASDP-Rosetta	OR135_ASDP-Rosetta_u		20	0.85	0.19	green	54.7	0.88	5.39	1.10	2.85	2.41	0	0.67	5.23	1.89	334	379	273	388	0	39.594	153	25	1	136	76	72	68	0
209	OR135	autonoe-Rosetta-alpha	OR135_autonoe-Rosetta-alpha_ur		9	1.01	0.52	green		0.85	5.50	1.46	0.38	0.28	0	0.67	7.96	2.07	0	0	0	0	0		0	0	0	0	136	99	82	0
210	OR135	Cheshire	OR135_Cheshire_s		1	1.29		green			1.16	0.70	8.29	-3.46	0	-1.22	0.49	-0.65	0	0	0	0	0		0	0	0	0	23	19	15	0
216	OR135	Cheshire-YAPP	OR135_Cheshire-YAPP_c		100	1.21	0.85	red		0.85	1.45	-4.73	16.59	-5.35	5.9	-3.11	-7.38	-6.27	0	0	0	0	0		0	0	0	0	5147	4596	4016	0
211	OR135	Cheshire-YAPP	OR135_Cheshire-YAPP_u		100	1.57	0.94	red		0.82	0.42	-1.80	6.90	-6.54	0.5	-3.27	-7.57	-6.15	0	0	0	0	0		0	0	0	0	5848	5233	4739	0
281	OR135	CS-HM-Rosetta	OR135_CS-HM-Rosetta_sr		20	1.01	0.74	green		0.85	5.67	1.30	1.15	0.68	0	0.75	7.52	1.95	0	0	0	0	0		0	0	0	0	239	181	141	0
205	OR135	CS-Rosetta	OR135_CS-Rosetta_s		5	0.93	0.59	green		0.88	5.35	1.12	2.56	0.83	0	0.87	8.36	2.25	0	0	0	0	0		0	0	0	0	28	18	13	0
212	OR135	CYANA	OR135_CYANA_c		20	0.75	0.38	green	75.7	0.91	4.03	0.78	4.17	-2.58	0	-0.47	-4.96	-2.13	626	659	584	966	0	74.336	20	0	0	0	359	277	198	0
198	OR135	CYANA	OR135_CYANA_u		20	0.98	0.38	green	66.3	0.89	3.55	0.88	1.70	-2.10	0	-0.35	-4.24	-1.71	509	524	464	733	0	74.119	46	0	0	0	377	292	228	0
226	OR135	Ponderosa	OR135_Ponderosa_r		20	1.91	0.28	orange		0.79	2.47	-2.79	27.68	0.93	0.3	0.87	-7.39	-2.48	300	392	184	209	0	25.484	896	637	507	298	774	719	647	0
204	OR135	UNIO	OR135_UNIO_r		20	1.01	0.56	orange	54.1	0.88	2.34	-2.06	23.08	-3.93	0	-0.71	-6.86	-4.08	353	438	249	403	0	51.397	75	19	9	226	620	472	377	0
203	OR135	UNIO	OR135_UNIO_u		20	0.98	0.60	orange	60.2	0.87	2.97	-3.84	28.58	-3.62	0.2	-0.55	-7.17	-3.67	395	396	354	508	0	63.971	63	21	17	225	743	623	510	0
OR36	OR36		OR36		20		0.77	green	60.2	0.91	4.65	-1.73	18.92	-1.71	0	0.90	-1.71	0.41	749	1080	824	1090	0	46.892	134	2	0	196	155	54	10	0
189	OR36	ARIA	OR36_ARIA_c		10	1.02	0.80	green	70.4	0.91	4.71	-1.23	17.38	-0.48	0	1.06	-1.11	0.71	909	2298	890	1105	1717	40.379	921	567	344	236	334	216	147	0
190	OR36	ARIA	OR36_ARIA_cr		9	1.04	0.72	green	71.5	0.91	4.55	-1.35	17.54	-2.19	0.2	0.35	-1.11	0.18	912	2292	856	1161	1740	51.904	674	392	229	236	496	329	243	165
332	OR36	ARIA	OR36_ARIA_ur	TRUE	10	11.74	11.09	green	73.9	0.70	-0.51		39.19	-5.78			-4.07		1267	1607	1340	1069	1771	35.997	10010	8509	7215	236	1658	1296	1028	165
192	OR36	ASDP-CNS	OR36_ASDP-CNS_c		20	1.54	0.98	green	57.1	0.90	3.90	-1.83	21.44	-2.16	0.3	0.39	-3.07	-0.65	586	734	537	673	0	84.888	701	50	2	246	1117	689	411	0
193	OR36	ASDP-CNS	OR36_ASDP-CNS_cr		20	1.38	0.96	green	56.5	0.90	4.38	-1.98	22.76	-1.62	0	0.75	-3.46	-0.53	591	737	542	662	0	80.217	569	39	0	244	855	530	309	165
178	OR36	ASDP-CNS	OR36_ASDP-CNS_u		20	1.35	1.00	green	50.7	0.88	3.96	-1.72	20.99	-2.03	0	0.63	-3.43	-0.59	590	683	520	514	0	63.010	577	34	2	246	1074	626	402	0
179	OR36	ASDP-CNS	OR36_ASDP-CNS_ur		20	1.33	0.91	green	48.2	0.87	4.38	-2.57	26.57	-1.90	0	0.43	-3.80	-0.89	591	684	502	540	0	74.609	629	83	3	246	1107	678	449	165
184	OR36	autonoe-Rosetta-alpha	OR36_autonoe-Rosetta-alpha_ur		10	1.46	1.24	green		0.89	5.26	0.82	3.98	-0.27	0	1.26	6.62	2.25	0	0	0	0	0		0	0	0	0	224	154	115	0
324	OR36	Cheshire	OR36_Cheshire_s		1	2.64		green			1.59			-1.95			-0.65		0	0	0	0	0		0	0	0	0	39	34	28	0
194	OR36	Cheshire-YAPP	OR36_Cheshire-YAPP_c		50	1.20	0.96	red		0.88	2.26	-3.39	32.86	-5.46	0.1	-1.02	-7.92	-4.55	0	0	0	0	0		0	0	0	0	4246	3669	3068	0
188	OR36	Cheshire-YAPP	OR36_Cheshire-YAPP_u		50	1.24	0.91	red		0.86	2.26	-4.20	23.87	-5.67	0.1	-1.26	-7.74	-5.03	0	0	0	0	0		0	0	0	0	4491	3840	3232	0
183	OR36	CS-DP-Rosetta	OR36_CS-DP-Rosetta_u		5	1.42	1.07	green		0.88	5.32	0.80	4.58	1.14	0	1.69	6.10	2.31	0	0	0	0	0		0	0	0	0	91	66	45	0
187	OR36	CYANA	OR36_CYANA_c		20	1.19	0.51	green	63.7	0.91	3.95	0.34	7.74	-2.08	0	0.16	-4.54	-1.42	774	1037	816	1200	0	85.234	73	0	0	0	786	576	432	0
170	OR36	CYANA	OR36_CYANA_u		20	1.14	0.33	green	58.0	0.85	3.14	-0.90	15.94	-3.26	0	0.12	-6.36	-2.66	907	956	860	1262	0	95.044	67	0	0	0	790	571	406	0
182	OR36	Ponderosa	OR36_Ponderosa_r		20	2.78	0.27	orange	30.7	0.78	3.43	-2.19	22.78	0.23	0	1.10	-7.78	-2.60	697	743	542	345	0	35.051	4669	3304	2390	193	996	433	270	0
180	OR36	UNIO	OR36_UNIO_r		20	1.57	1.06	green	36.1	0.88	1.89	0.78	4.61	-3.29	0.3	-0.35	-6.22	-3.08	331	608	221	253	0	31.632	239	59	29	372	1161	942	757	0
181	OR36	UNIO	OR36_UNIO_u		20	1.43	0.70	orange	47.6	0.86	2.19	-0.80	14.94	-3.37	0	-0.39	-7.31	-3.90	773	600	708	872	0	73.111	589	307	249	360	1159	922	740	0

EntryID	Target	Program Type	Entry	Invalid	Conformers	Accuracy	Convergence	ROG	NOE completeness	DP Score	WI packing quality	MP Clash Z-score	MP Clash score (pdb style)	WI Ramachandran appearance	MP % Ramachandran disallowed	PC_NMR ϕ/ψ Z-score	WI χ Z-	PC_NMR dihedral Z-score	Intra-NOEs	Seq-NOEs	Med-NOEs	Long-NOEs	Ambig-NOEs	Queen Information	NOE violations > 0.1 Å	NOE violations > 0.3 Å	NOE violations > 0.5 Å	dihedrals	dihedral violations > 1°	dihedral violations > 3°	dihedral violations > 5°	RDCs
StT322	StT322		StT322		20		0.57	green	63.4	0.81	2.39	-0.06	12.63	-4.23	0	-2.91	-3.68	-4.02	280	320	137	371	0	32.415	252	1	0	70	28	0	0	0
225	StT322	ARIA	StT322_ARIA_c		10	2.19	0.51	green	52.0	0.73	3.13	-1.08	17.35	-1.60	0.2	-2.12	-0.58	-2.66	218	694	111	232	393	31.127	249	148	87	78	244	128	97	0
333	StT322	ARIA	StT322_ARIA_u	TRUE	10	9.27	2.02	orange	62.4	0.51	-4.23		63.77	-5.92			-5.54		742	741	716	1789	2074	105.501	13546	11966	10478	78	557	364	254	0
229	StT322	ASDP-CNS	StT322_ASDP-CNS_c		20	1.91	0.67	red	62.7	0.80	1.05	-0.75	19.92	-2.66	0.1	-2.71	-7.79	-6.62	213	284	105	258	0	43.591	42	11	1	92	368	171	91	0
222	StT322	ASDP-CNS	StT322_ASDP-CNS_u		20	2.85	1.43	red	48.8	0.77	0.07	-1.08	21.45	-2.71	0	-1.49	-7.76	-5.14	237	247	79	143	0	39.702	52	2	0	92	399	249	155	0
230	StT322	ASDP-Rosetta	StT322_ASDP-Rosetta_c		20	1.49	0.81	green	61.5	0.81	2.48	-0.71	17.73	-0.50	0	-1.30	3.58	-1.06	213	284	105	258	0	43.591	175	52	10	92	266	195	130	0
223	StT322	ASDP-Rosetta	StT322_ASDP-Rosetta_u		20	2.28	1.50	green	48.0	0.79	1.96	0.03	9.89	0.66	0	-0.47	5.15	0.06	237	247	79	143	0	39.702	189	75	14	92	275	225	177	0
220	StT322	autonoe-Rosetta-alpha	StT322_autonoe-Rosetta-alpha_u		10	4.34	0.44	green			1.69	1.41	0.00	0.62	1.7	-1.30	5.83	-0.06	0	0	0	0	0		0	0	0	0	116	104	84	0
231	StT322	Cheshire	StT322_Cheshire_s		10	1.30	0.82	green		0.70	4.47	0.65	4.39	-3.14	0.5	-2.40	0.01	-2.13	0	0	0	0	0		0	0	0	0	190	174	158	0
224	StT322	CYANA	StT322_CYANA_c		20	2.83	0.50	orange	60.6	0.77	1.73	0.79	6.41	-3.34	1.1	-3.38	-6.44	-5.32	278	296	132	329	0	38.716	7	0	0	0	337	294	238	0
221	StT322	Ponderosa	StT322_Ponderosa_r		20	3.69	0.25	orange		0.59	-0.14	-2.12	32.37	-0.38	0.1	-1.46	-5.92	-4.02	231	178	74	81	0	14.679	74	0	0	211	329	286	244	0
YR313A	YR313A		YR313A		20		0.97	green	61.2	0.87	2.70	-0.58	12.18	-2.87	0	-0.55	-1.31	-0.59	631	743	413	754	0	49.414	74	1	0	122	130	37	3	0
253	YR313A	ARIA	YR313A_ARIA_c		10	1.12	0.50	green	62.0	0.84	2.93	-0.93	16.27	-1.25	1	-0.63	-0.77	-0.83	608	1495	354	655	575	28.782	372	230	105	174	163	115	78	0
254	YR313A	ARIA	YR313A_ARIA_cr		10	1.39	0.64	green	61.3	0.83	2.42	-1.03	16.66	-3.21	0	-1.26	-0.49	-1.24	602	1500	346	616	545	47.096	386	218	123	174	417	235	154	112
334	YR313A	ARIA	YR313A_ARIA_ur	TRUE	10	14.15	12.74	orange	68.4	0.59	-2.20		34.61	-5.83			-4.20		822	1308	454	176	519	26.691	3931	3217	2629	174	1113	840	643	112
315	YR313A	ASDP-CNS	YR313A_ASDP-CNS_c		20	1.81	0.74	green	54.6	0.80	2.33	-2.64	27.03	-2.08	0.1	-1.06	-2.78	-2.01	478	555	297	484	0	79.991	105	4	0	198	412	146	51	0
276	YR313A	ASDP-CNS	YR313A_ASDP-CNS_u		19	1.44	1.11	green	51.4	0.84	2.48	-1.42	20.02	-2.24	0.1	-0.55	-3.12	-1.36	506	529	297	455	0	60.701	124	7	0	198	519	202	99	0
316	YR313A	ASDP-Rosetta	YR313A_ASDP-Rosetta_c		20	1.75	1.06	green	60.0	0.83	2.82	-0.18	10.02	-0.19	0	0.20	4.65	1.06	487	558	301	475	0	77.327	259	82	25	198	211	149	103	0
277	YR313A	ASDP-Rosetta	YR313A_ASDP-Rosetta_u		20	1.45	0.75	green	48.3	0.84	2.97	-0.11	9.98	-0.03	0.1	0.12	4.07	0.83	506	529	297	455	0	60.701	395	306	241	198	139	90	66	0
245	YR313A	autonoe-Rosetta-alpha	YR313A_autonoe-Rosetta-alpha_ur		10	1.20	0.75	green			3.83	0.83	3.82	-0.90	0	-0.04	6.40	1.30	0	0	0	0	0		0	0	0	0	169	138	112	0
327	YR313A	Cheshire	YR313A_Cheshire_s		1	2.04		green			-1.21			-3.28			-1.84		0	0	0	0	0		0	0	0	0	40	34	31	0
328	YR313A	Cheshire-YAPP	YR313A_Cheshire-YAPP_c		50	1.12	0.80	red		0.86	1.10		31.19	-5.86			-7.11		0	0	0	0	0		0	0	0	0	2989	2628	2301	0
261	YR313A	Cheshire-YAPP	YR313A_Cheshire-YAPP_u		100	1.73	1.53	orange		0.82	1.14	-6.95	21.30	-3.19	1.5	-0.63	-7.88	-4.44	0	0	0	0	0		0	0	0	0	1605	1123	821	0
282	YR313A	CS-HM-Rosetta	YR313A_CS-HM-Rosetta_sr		1	2.17		orange			-0.37	0.64	5.19	-2.72	0	-1.02	6.03	0.06	0	0	0	0	0		0	0	0	0	31	22	16	0
260	YR313A	CYANA	YR313A_CYANA_c		20	1.21	0.67	green	64.3	0.86	1.84	0.96	3.70	-2.88	0.3	-1.46	-4.65	-2.78	626	724	389	778	0	82.907	51	0	0	0	545	424	338	0
228	YR313A	Ponderosa	YR313A_Ponderosa_r		20	1.67	0.12	green		0.78	2.23	-3.21	27.18	-0.80	0	-0.43	-6.48	-2.90	638	723	426	737	0	73.187	14	2	0	412	586	523	440	0
237	YR313A	UNIO	YR313A_UNIO_r		20	2.94	1.81	red	40.7	0.82	-0.24	-2.71	24.38	-4.85	0.1	-1.46	-7.07	-4.49	291	529	102	192	0	27.243	264	77	47	314	1315	1177	1031	0
