# Supplementary material for: Analysis of the structural quality of the CASD-NMR 2013 entries
Source: J Biomol NMR. 2015 Jun 3;62(4):527–40. doi: 10.1007/s10858-015-9949-0 (PMC4569653; doi:10.1007/s10858-015-9949-0)
Supplement: Supplementary file 2 — Supplementary material 2 (PDF 5022 kb) [file 10858_2015_9949_MOESM2_ESM.pdf]

# Analysis of the Structural Quality of the CASD-NMR 2013 Entries

# Supplementary materials

Timothy J. Ragan[1&], Rasmus H. Fogh[1&], Roberto Tejero[2], Wim Vranken[3], Gaetano T. Montelione[4], Antonio Rosato[5] and Geerten W. Vuister[1*]

[1] Department of Biochemistry, School of Biological Sciences, University of Leicester, Henry Wellcome building, Lancaster Road, Leicester, LE1 9HN, United Kingdom.

[2] Departamento de Química Física, Universidad de Valencia, Avda. Dr. Moliner 50. 46100 Burjassot (Valencia) Spain.

[3] Structural Biology Brussels, Pleinlaan 2, Vrije Universiteit Brussel and (IB)² Interuniversity Institute of Bioinformatics in Brussels, ULB-VUB, Triomflaan, 1050 Brussels, Belgium.

[4] Center for Advanced Biotechnology and Medicine, Department of Molecular Biology and Biochemistry, and Northeast Structural Genomics Consortium, Rutgers, The State University of New Jersey, and Robert Wood Johnson Medical School, Piscataway, NJ 08854.

[5] Magnetic Resonance Center, Department of Chemistry, University of Florence, 50019, Sesto Fiorentino, Italy.

[&] Equal contributions

[*] To whom correspondence should be addressed (gv29@le.ac.uk)

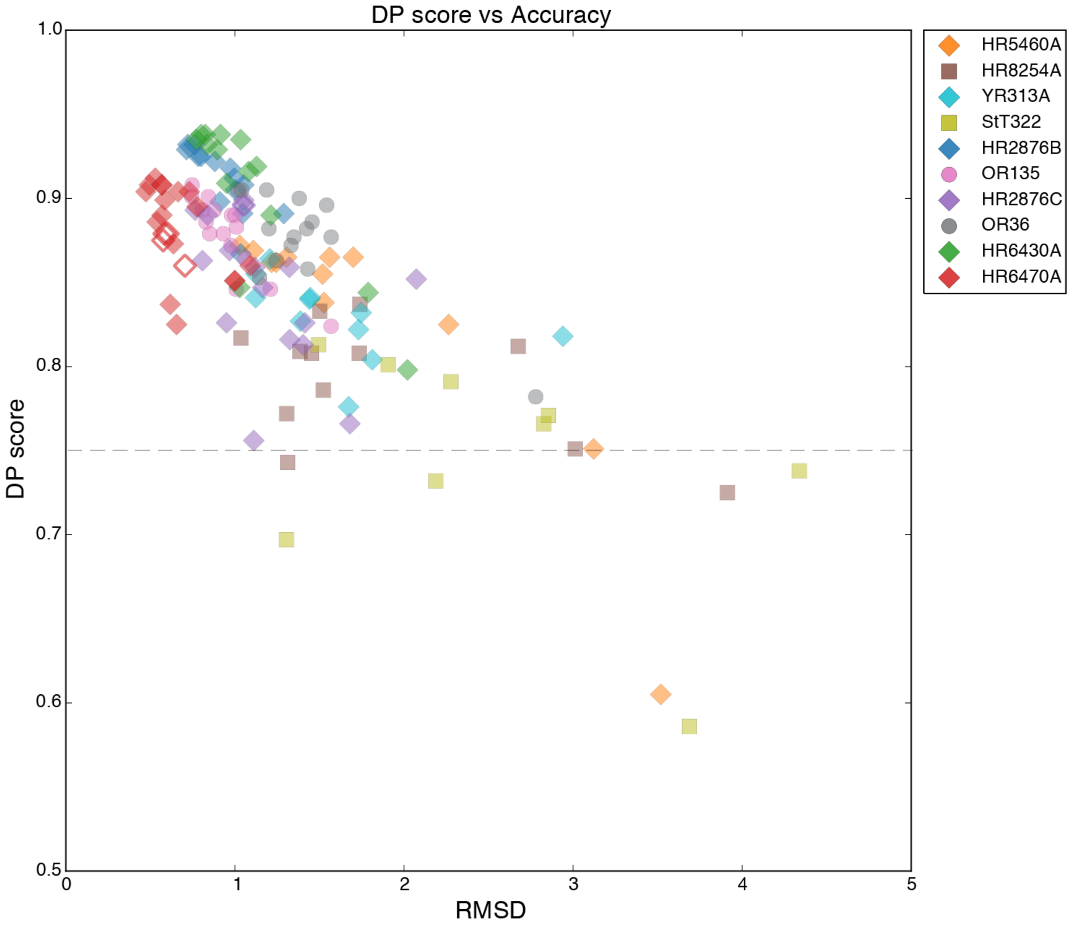


Supplementary Figure 1. DP score *vs* Accuracy. The DP score *vs* mean all by all pairwise backbone RMSD to the reference structure is plotted for all valid entries. The dashed line indicates the more stringent lower threshold of 0.75 for identifying reliable (RMSDs to reference < ~3 Å) models. Symbols for each target are indicated on the right. Open symbols indicate entries generated from truncated input sequences.

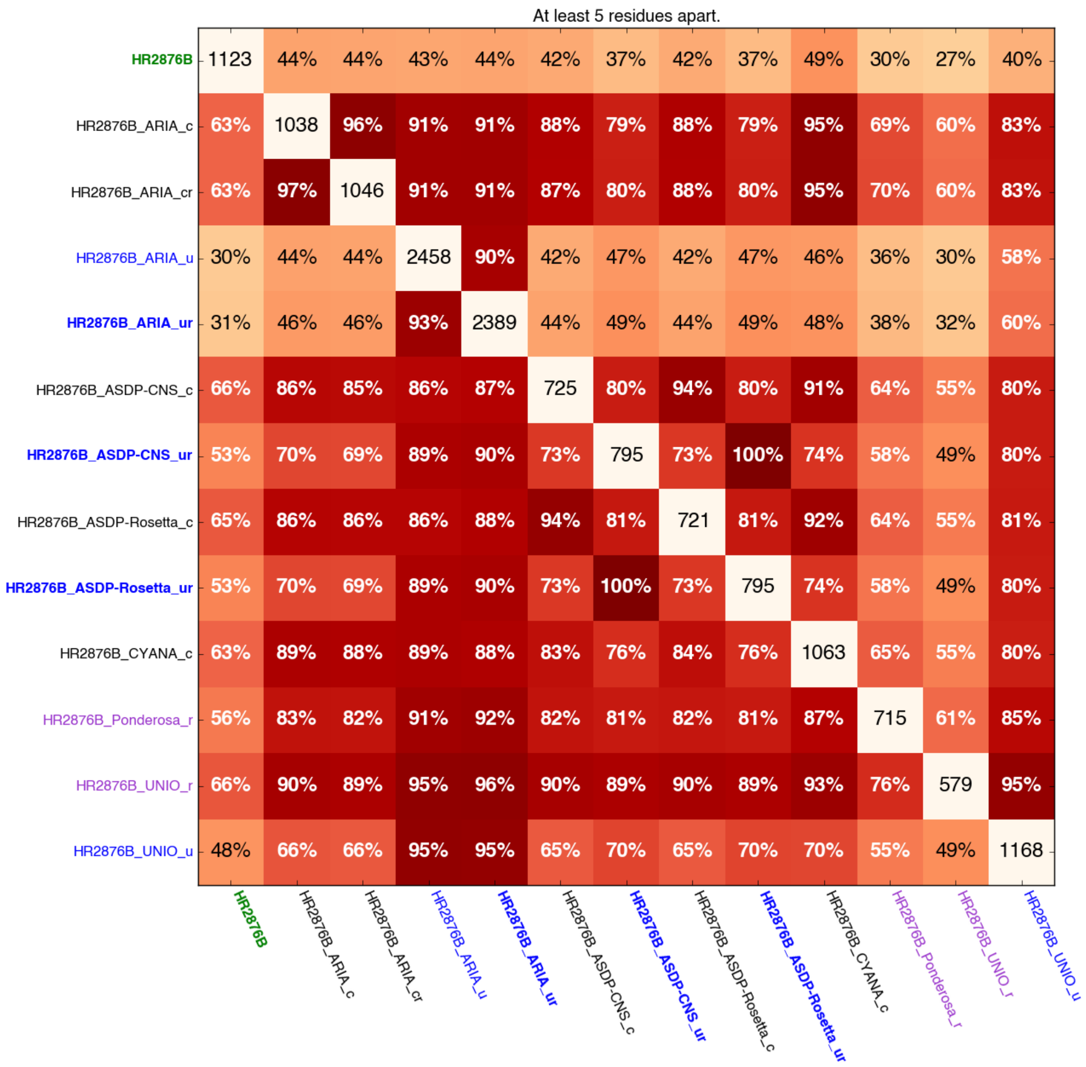


Supplementary Figure 2A. Heatmap of the fractions of overlapping long-range NOE restraints between the HR2876B target and entries, determined on the basis of pseudo-atom.

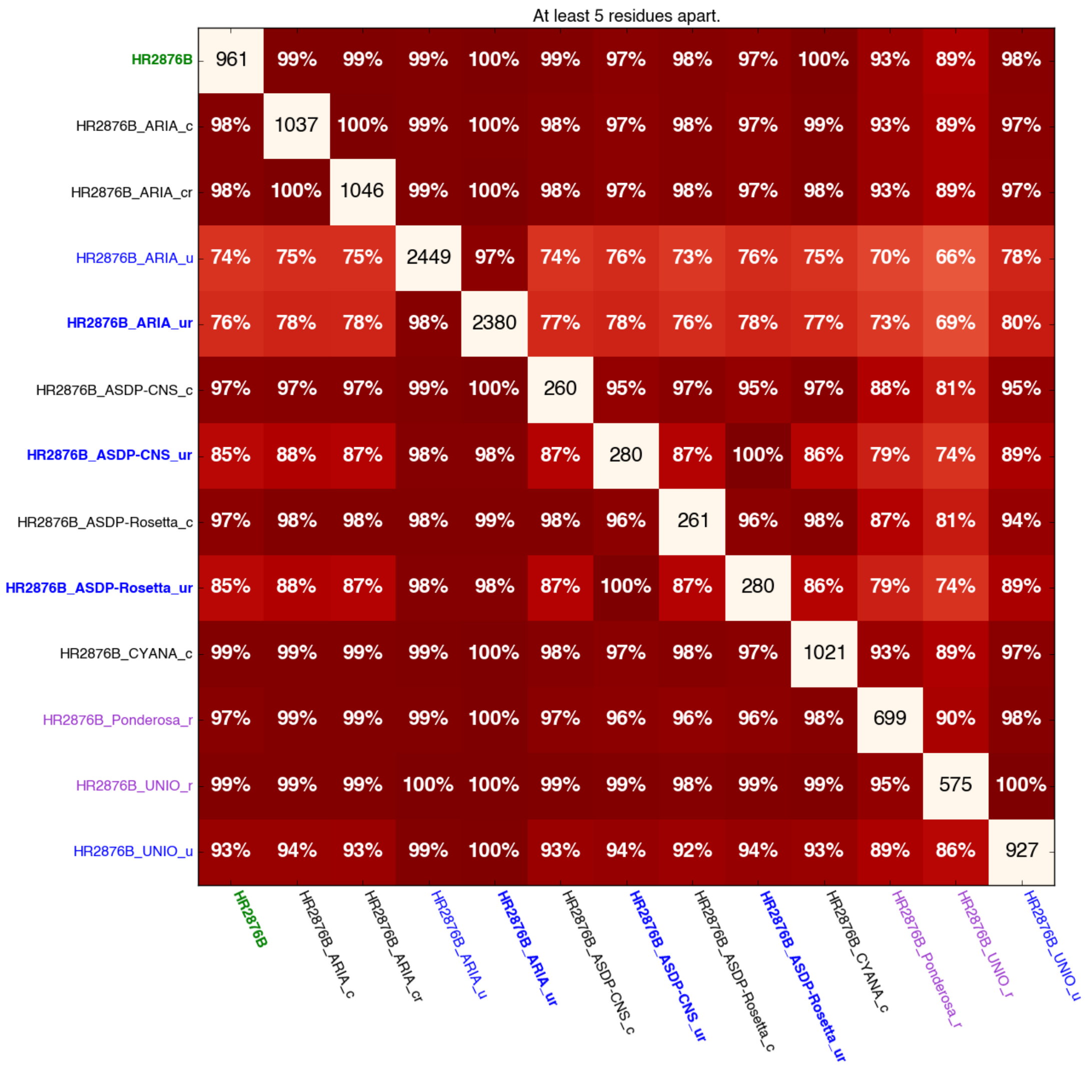


Supplementary Figure 2B. Heatmap of the fractions of overlapping long-range NOE restraints between the HR2876B target and entries, determined on the basis of residue.

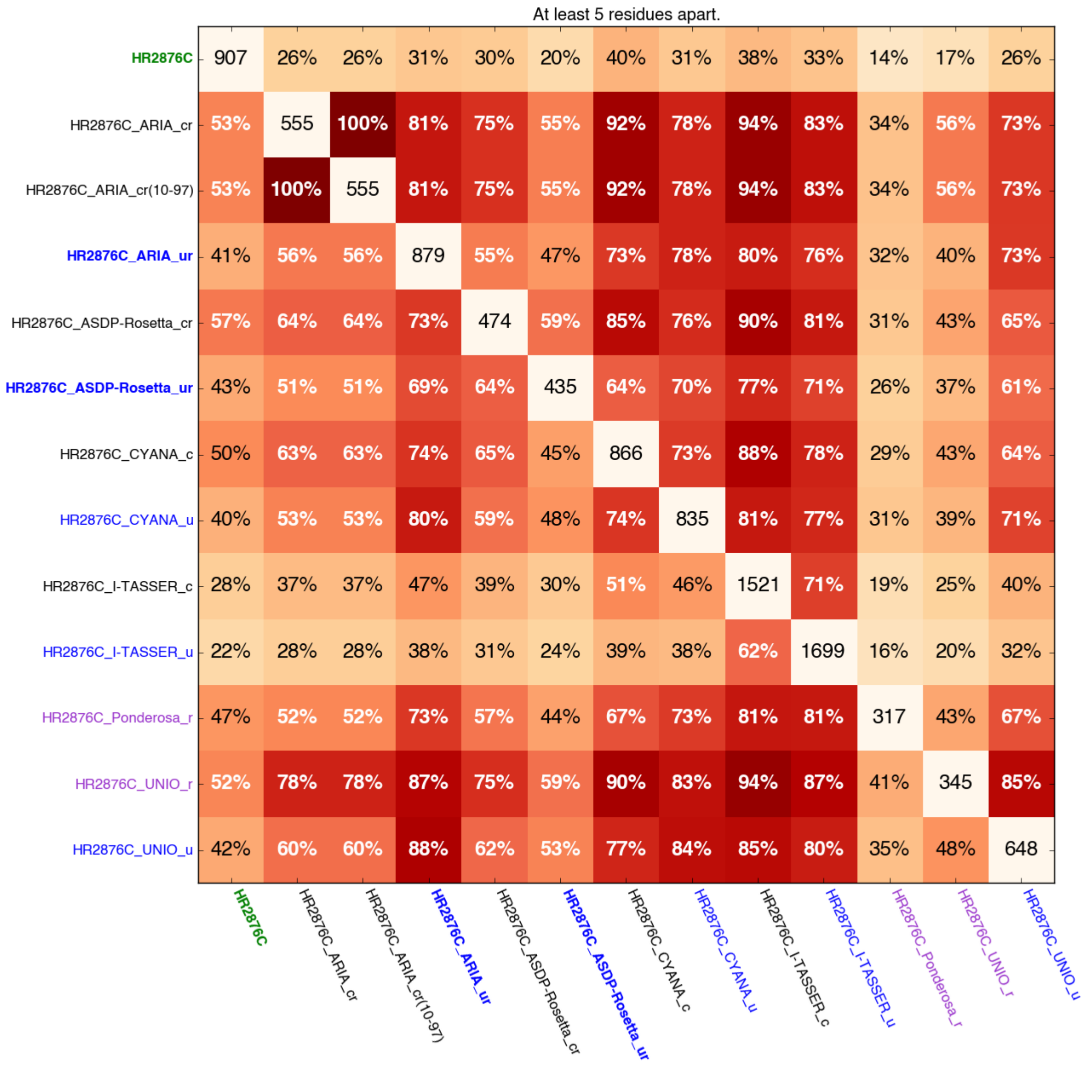


Supplementary Figure 3A. Heatmap of the fractions of overlapping long-range NOE restraints between the HR2876C target and entries, determined on the basis of pseudo-atom.

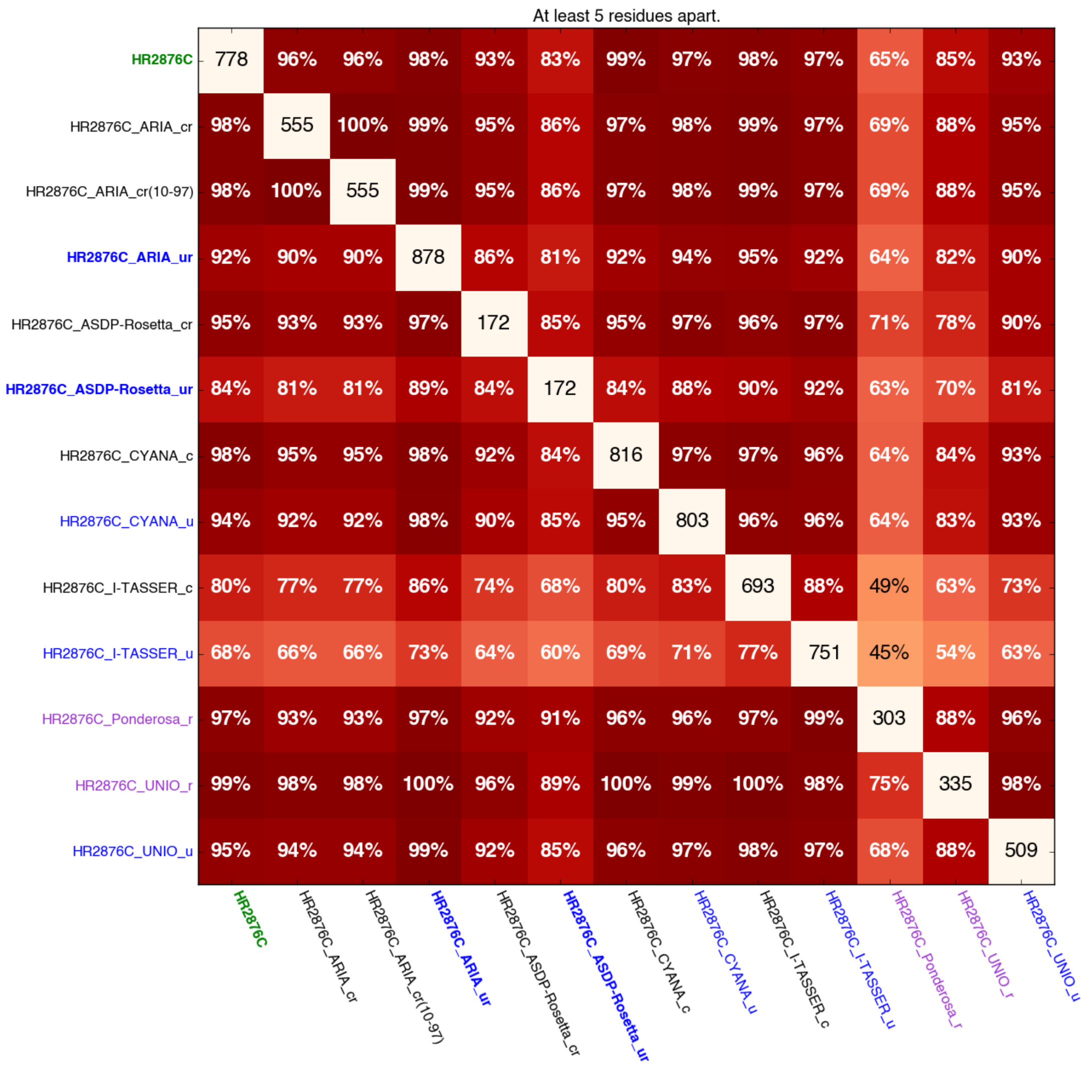


Supplementary Figure 3B. Heatmap of the fractions of overlapping long-range NOE restraints between the HR2876C target and entries, determined on the basis of residue.

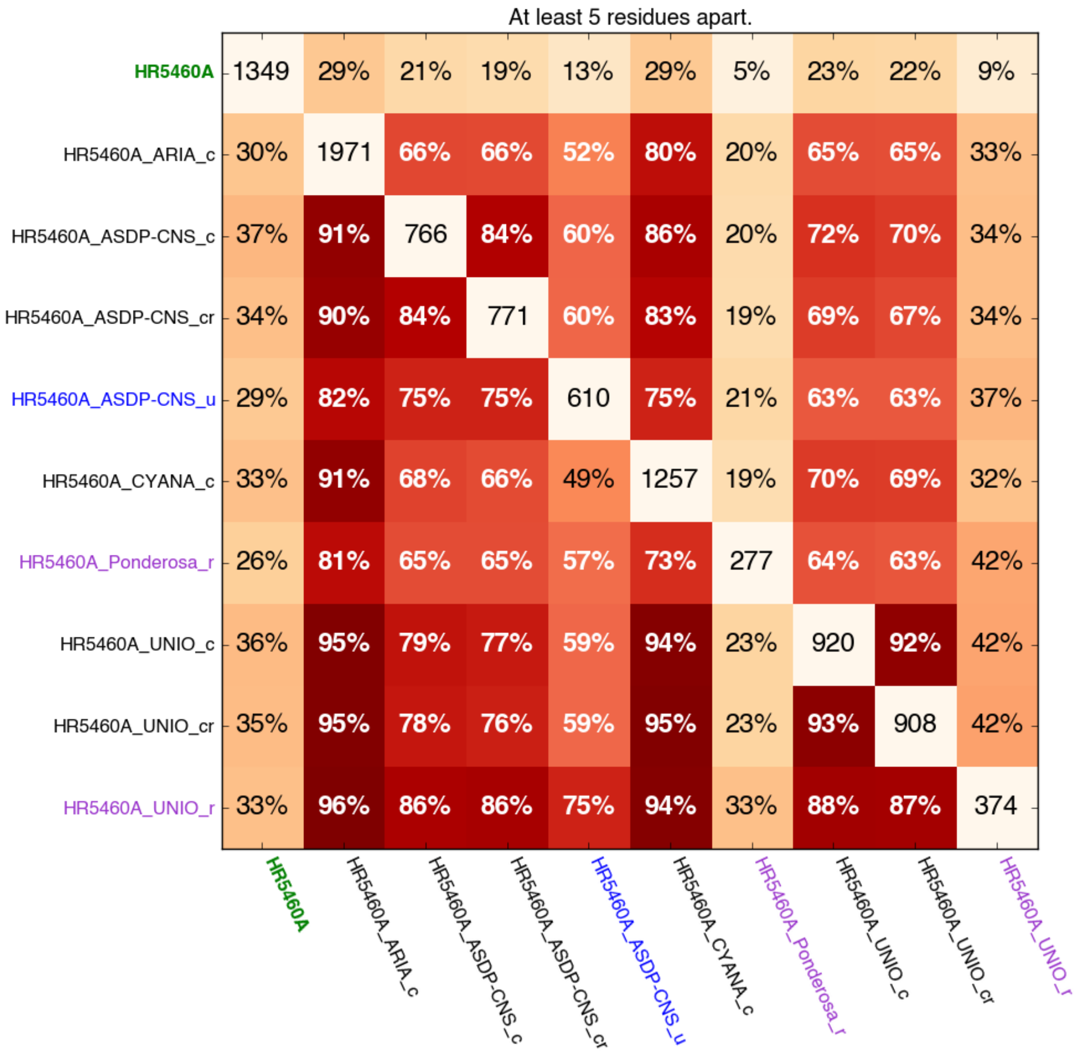


Supplementary Figure 4A. Heatmap of the fractions of overlapping long-range NOE restraints between the HR5460A target and entries, determined on the basis of pseudo-atom.

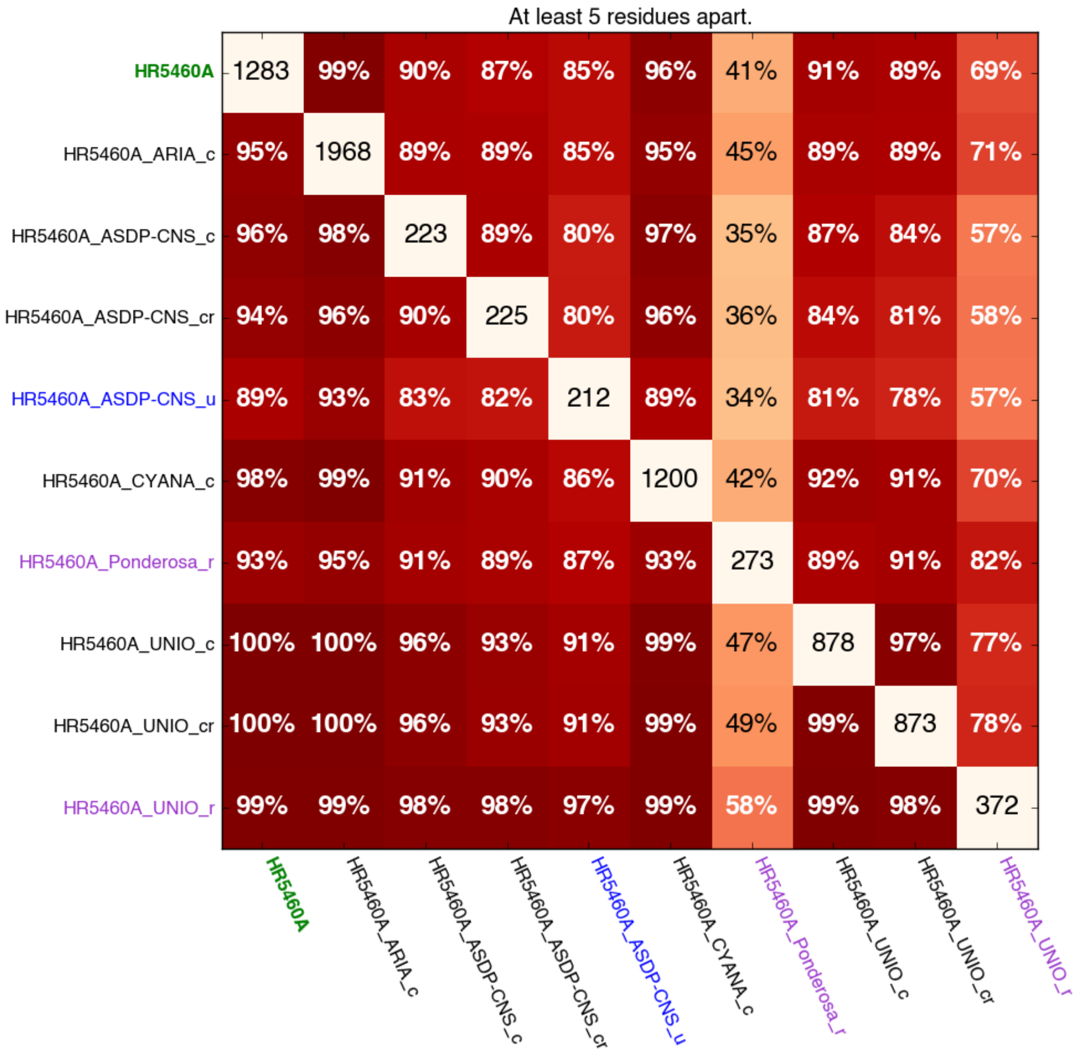


Supplementary Figure 4B. Heatmap of the fractions of overlapping long-range NOE restraints between the HR5460A target and entries, determined on the basis of residue.

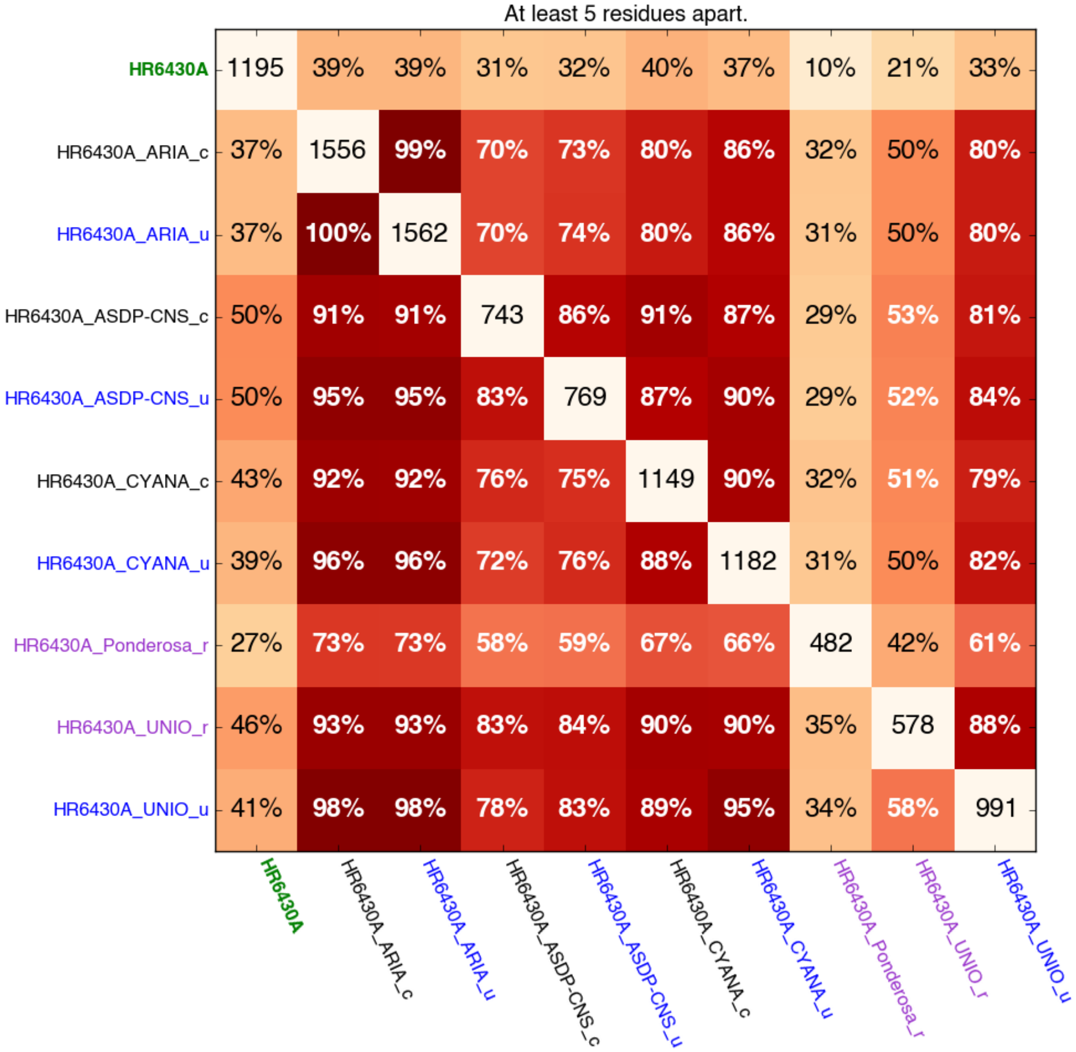


Supplementary Figure 5A. Heatmap of the fractions of overlapping long-range NOE restraints between the HR6430A target and entries, determined on the basis of pseudo-atom.

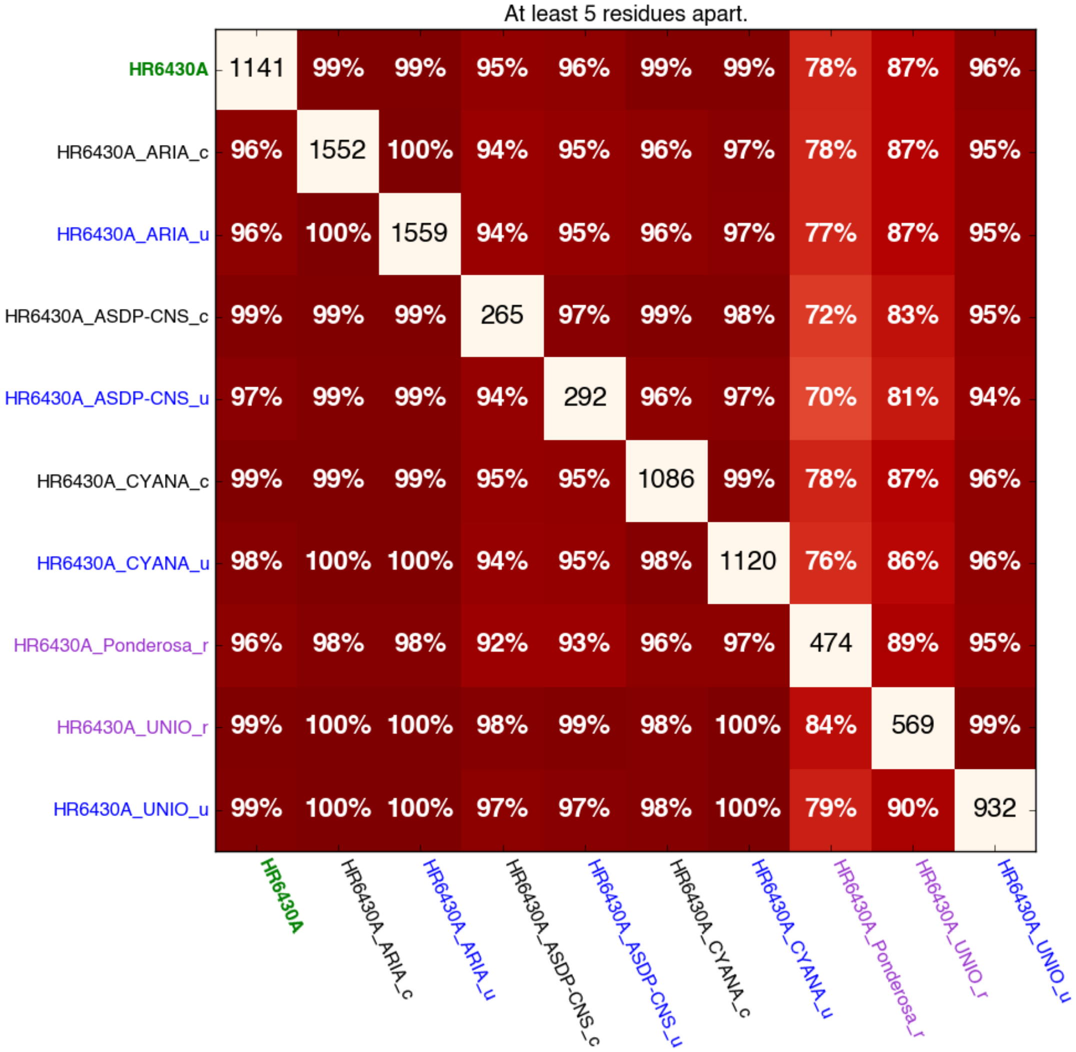


Supplementary Figure 5B. Heatmap of the fractions of overlapping long-range NOE restraints between the HR6430A target and entries, determined on the basis of residue.

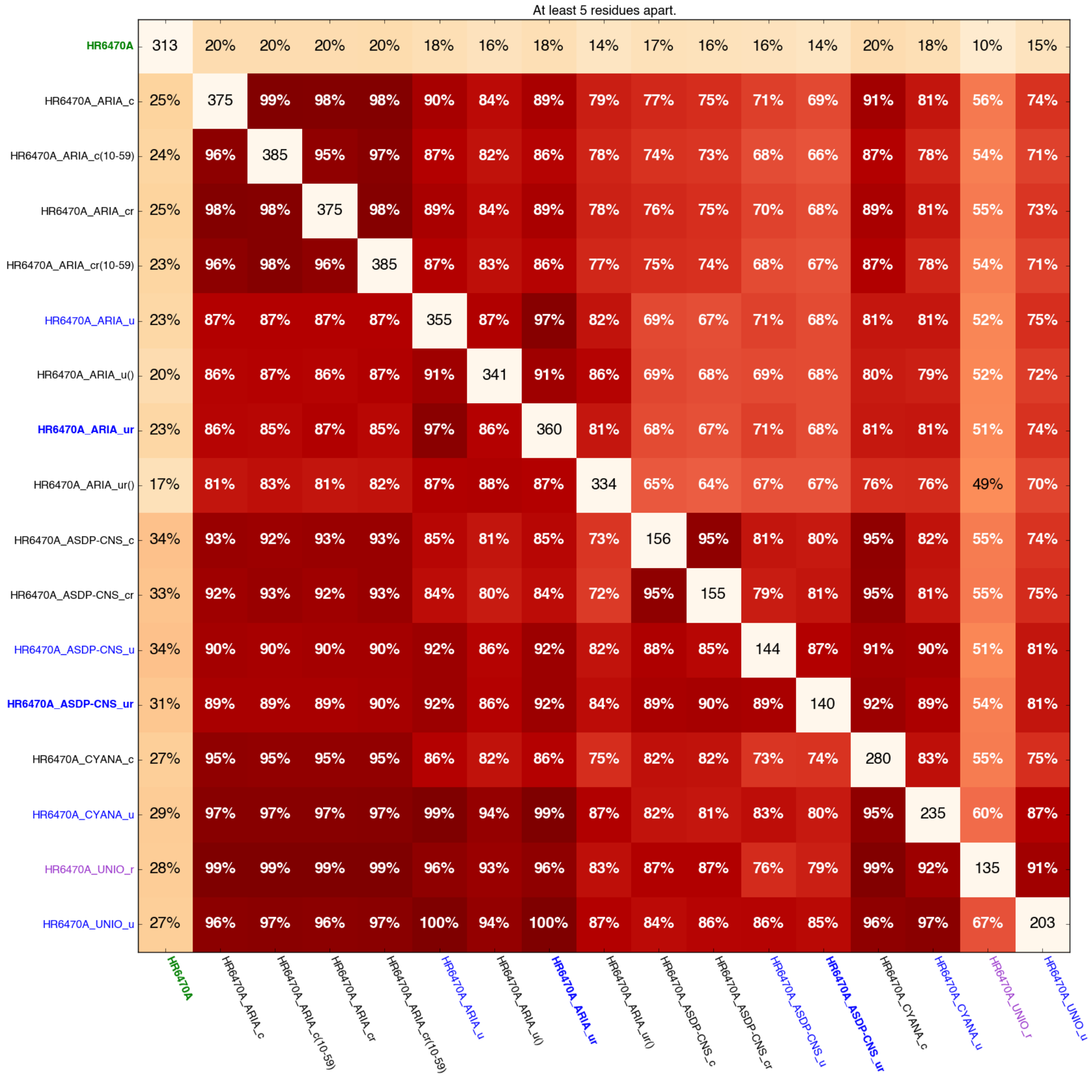


Supplementary Figure 6A. Heatmap of the fractions of overlapping long-range NOE restraints between the HR6470A target and entries, determined on the basis of pseudo-atom.

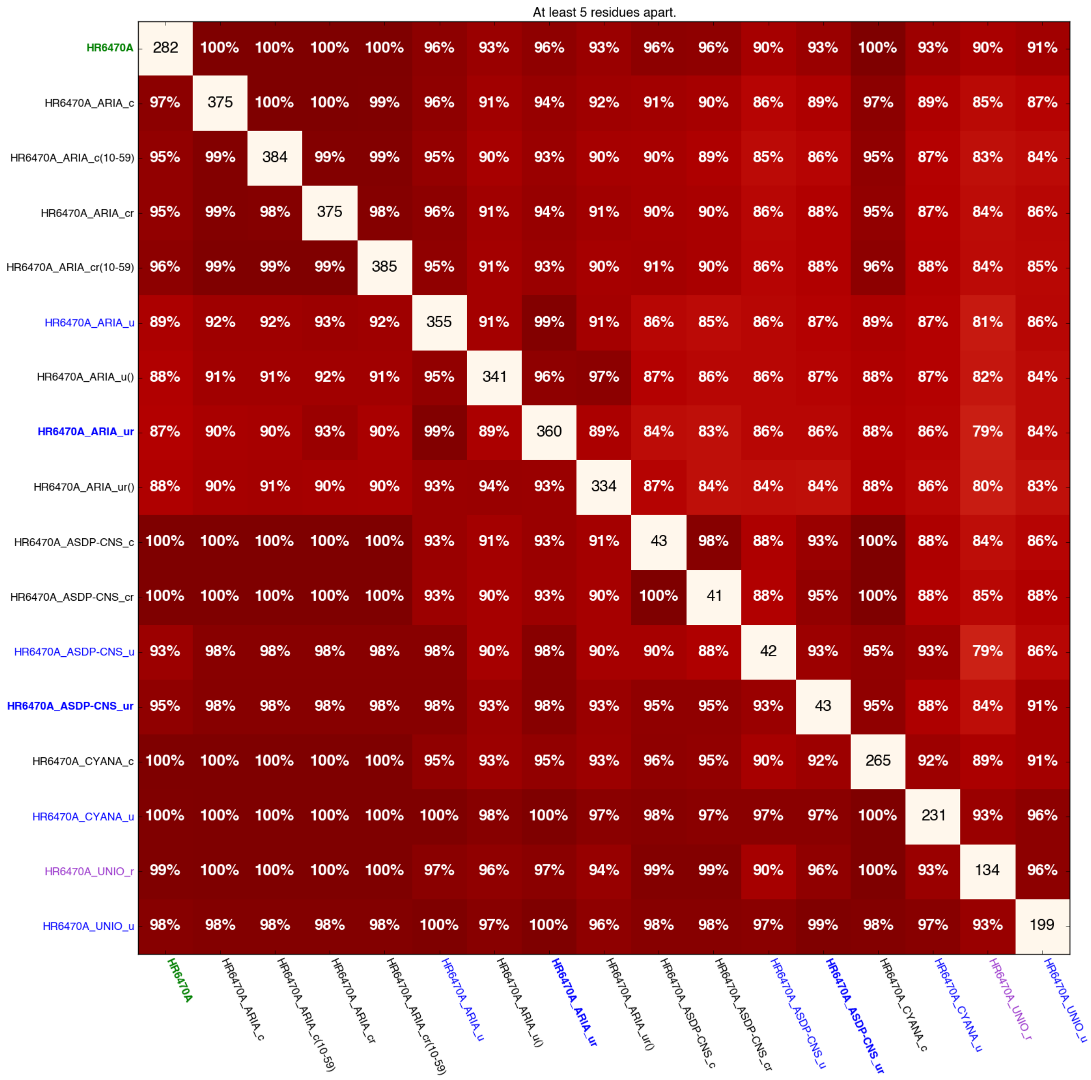

Supplementary Figure 6B. Heatmap of the fractions of overlapping long-range NOE restraints between the HR6470A target and entries, determined on the basis of residue.

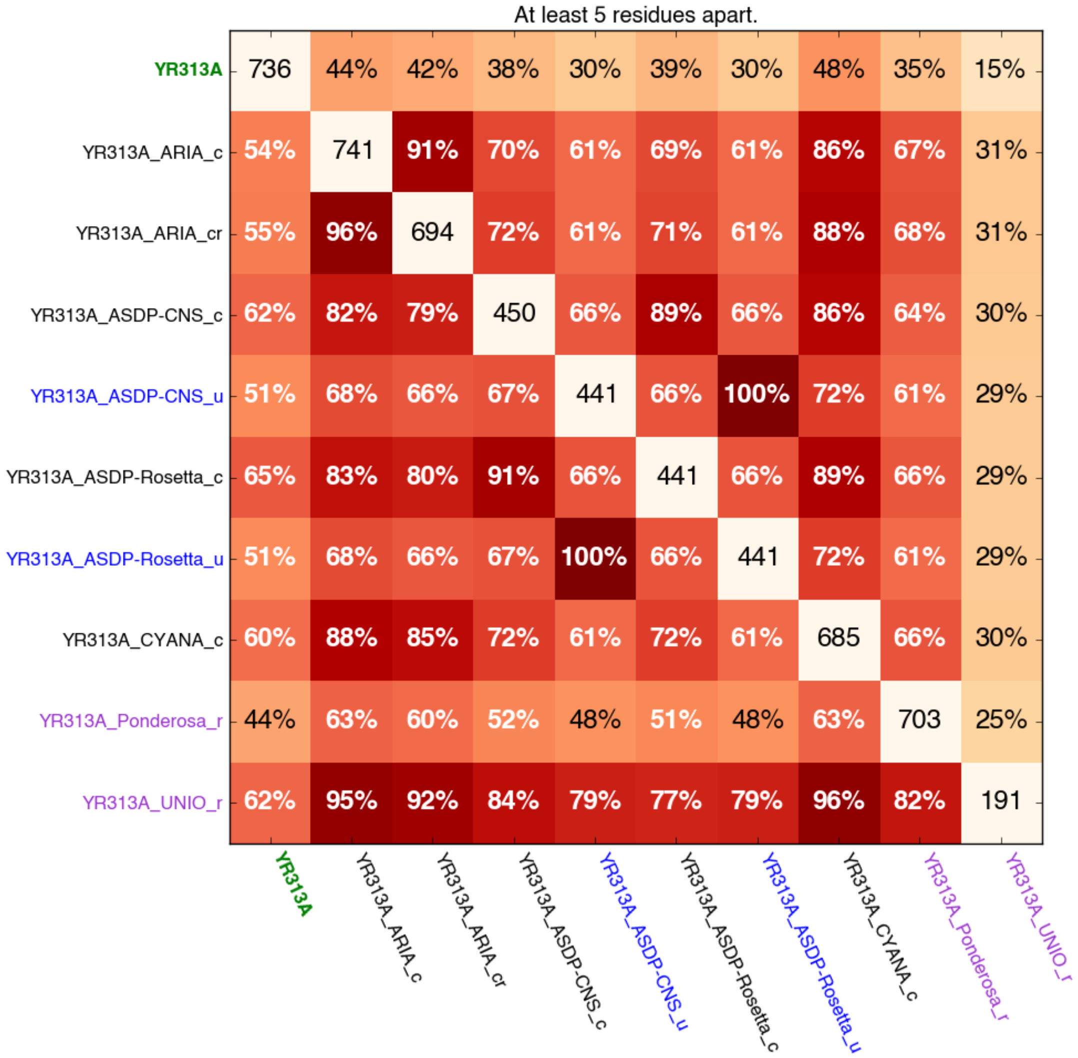


Supplementary Figure 7A. Heatmap of the fractions of overlapping long-range NOE restraints between the YR313A target and entries, determined on the basis of pseudo-atom.

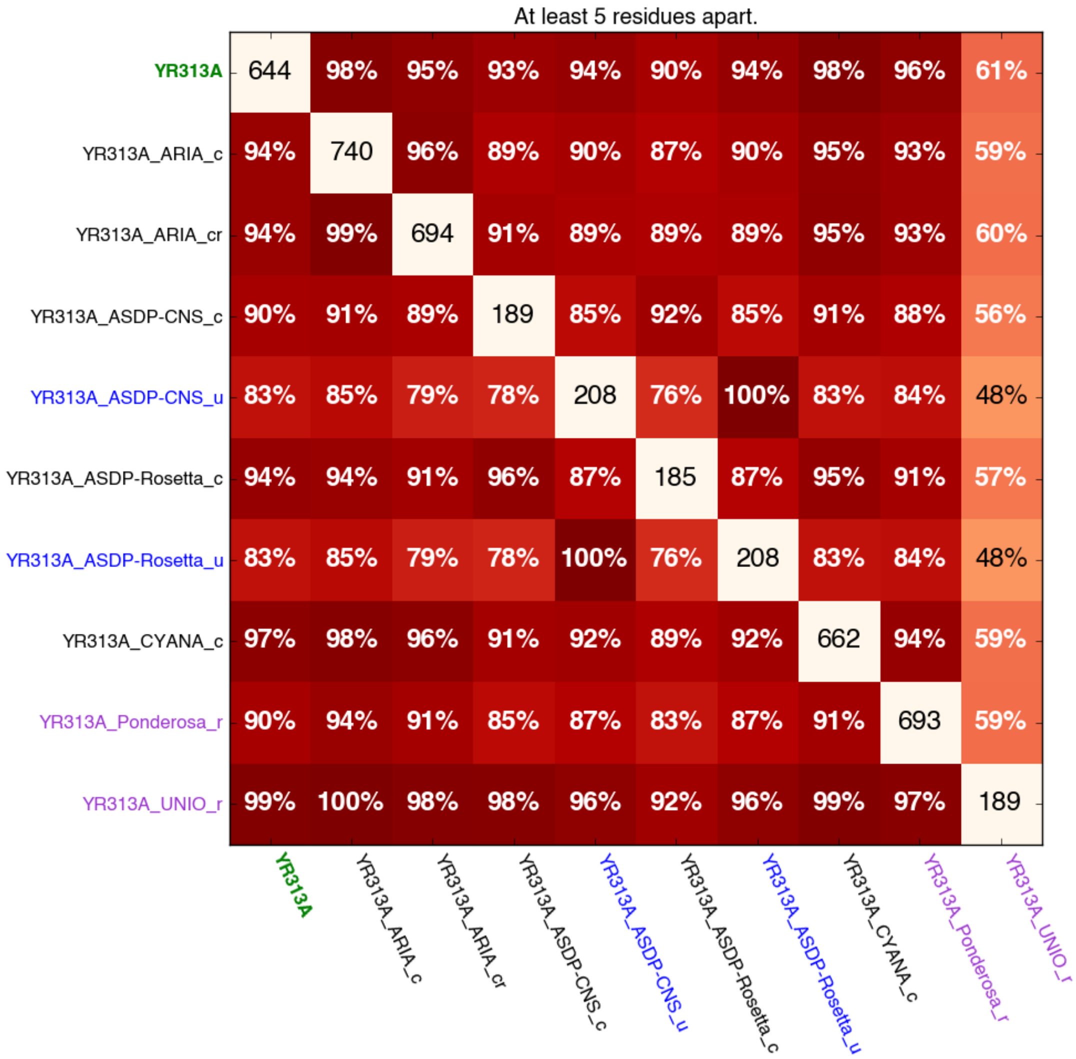


Supplementary Figure 7B. Heatmap of the fractions of overlapping long-range NOE restraints between the YR313A target and entries, determined on the basis of residue.

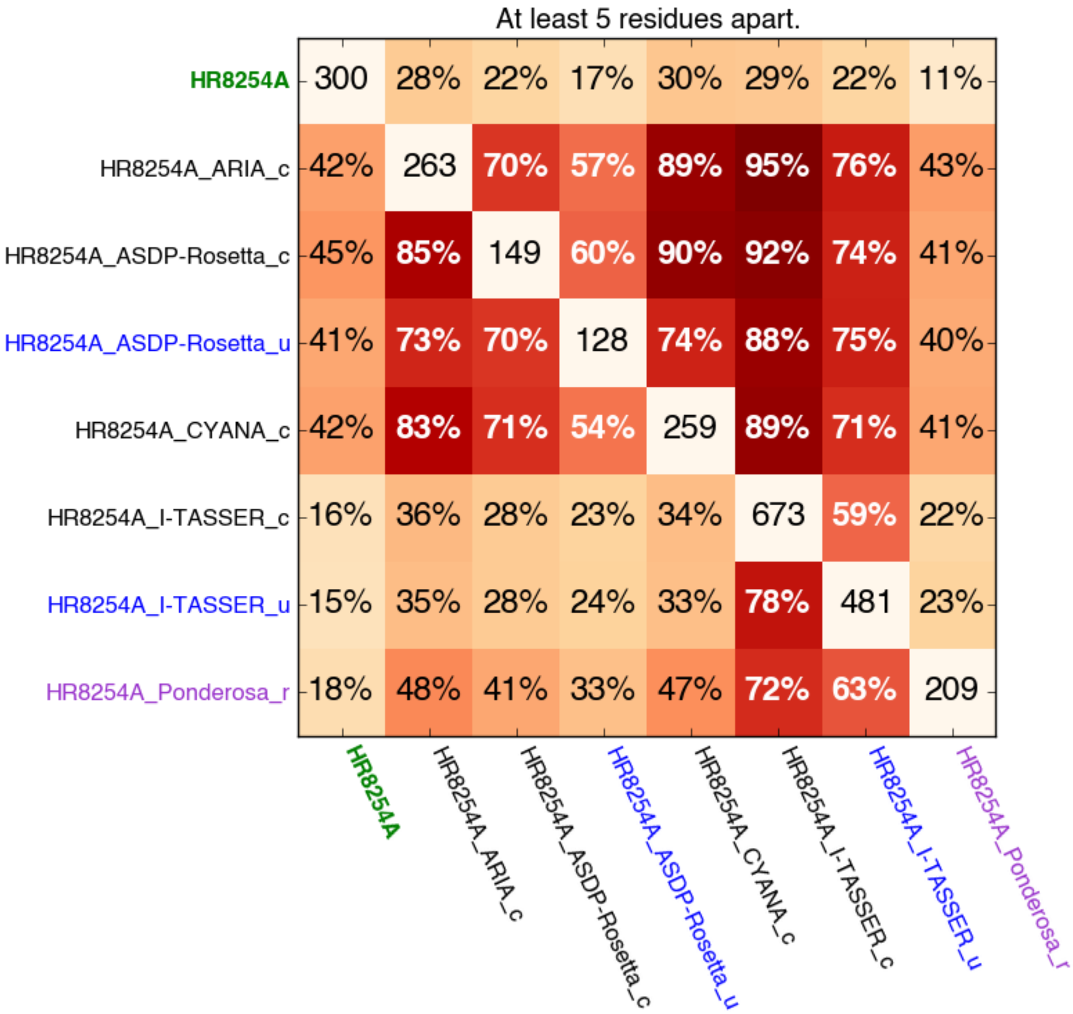


Supplementary Figure 8A. Heatmap of the fractions of overlapping long-range NOE restraints between the HR8254A target and entries, determined on the basis of pseudo-atom.

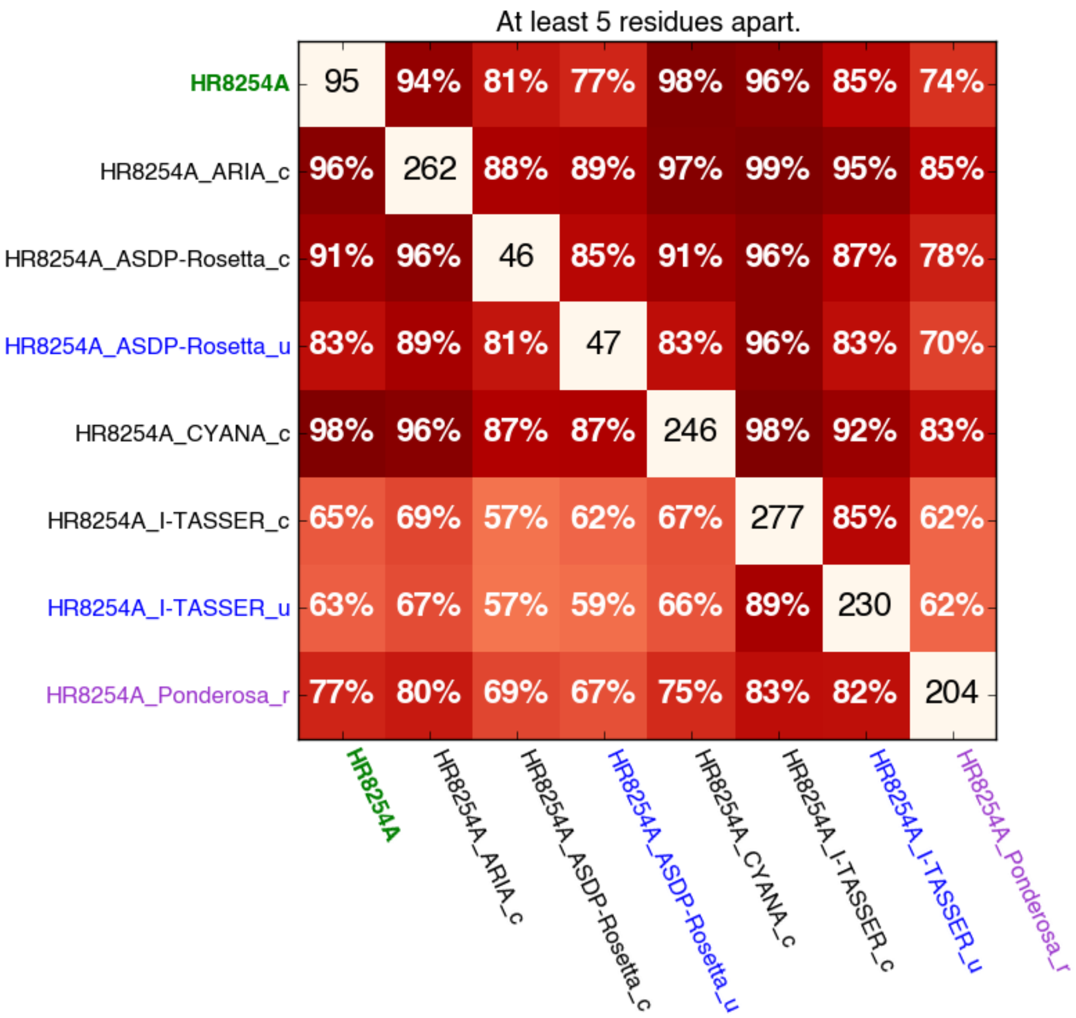


Supplementary Figure 8B. Heatmap of the fractions of overlapping long-range NOE restraints between the HR8254A target and entries, determined on the basis of residue.

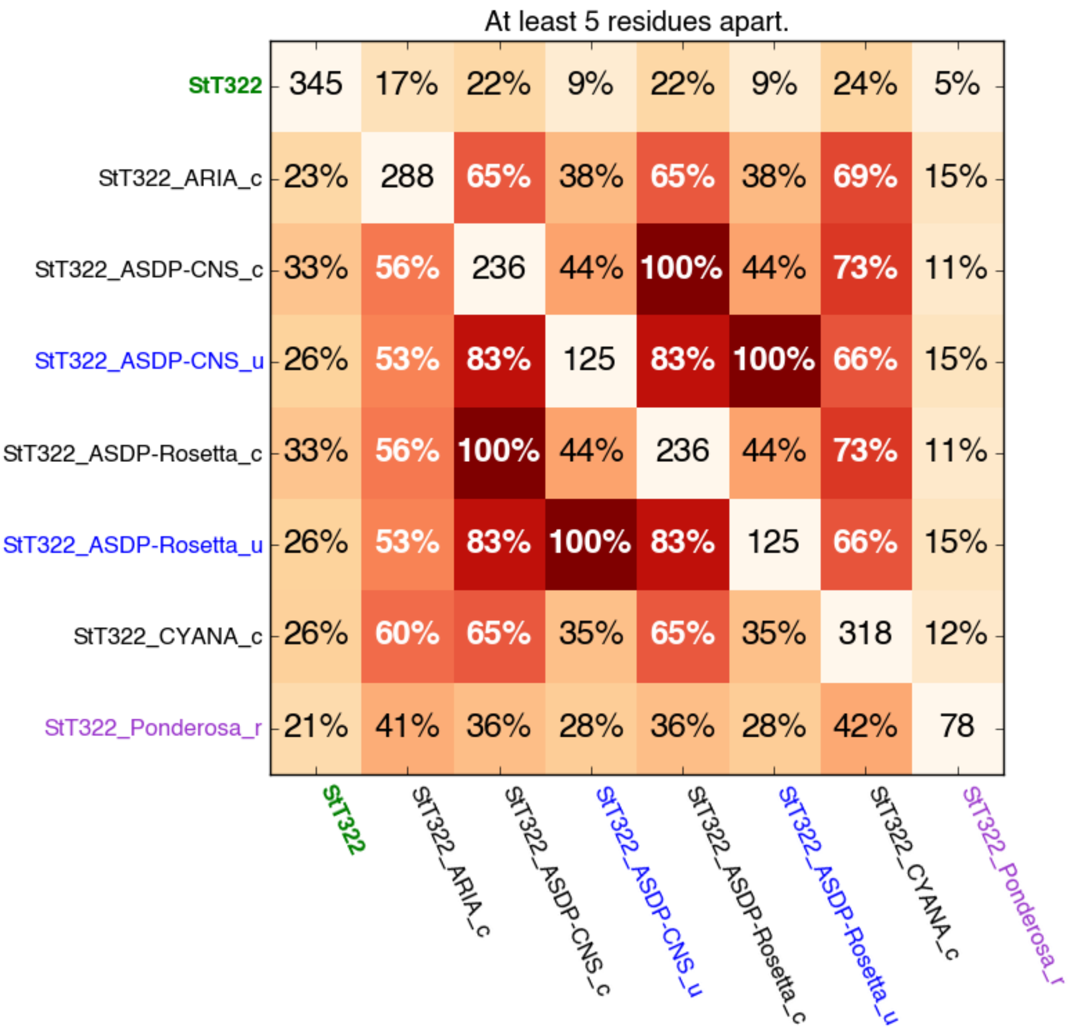


Supplementary Figure 9A. Heatmap of the fractions of overlapping long-range NOE restraints between the StT322 target and entries, determined on the basis of pseudo-atom.

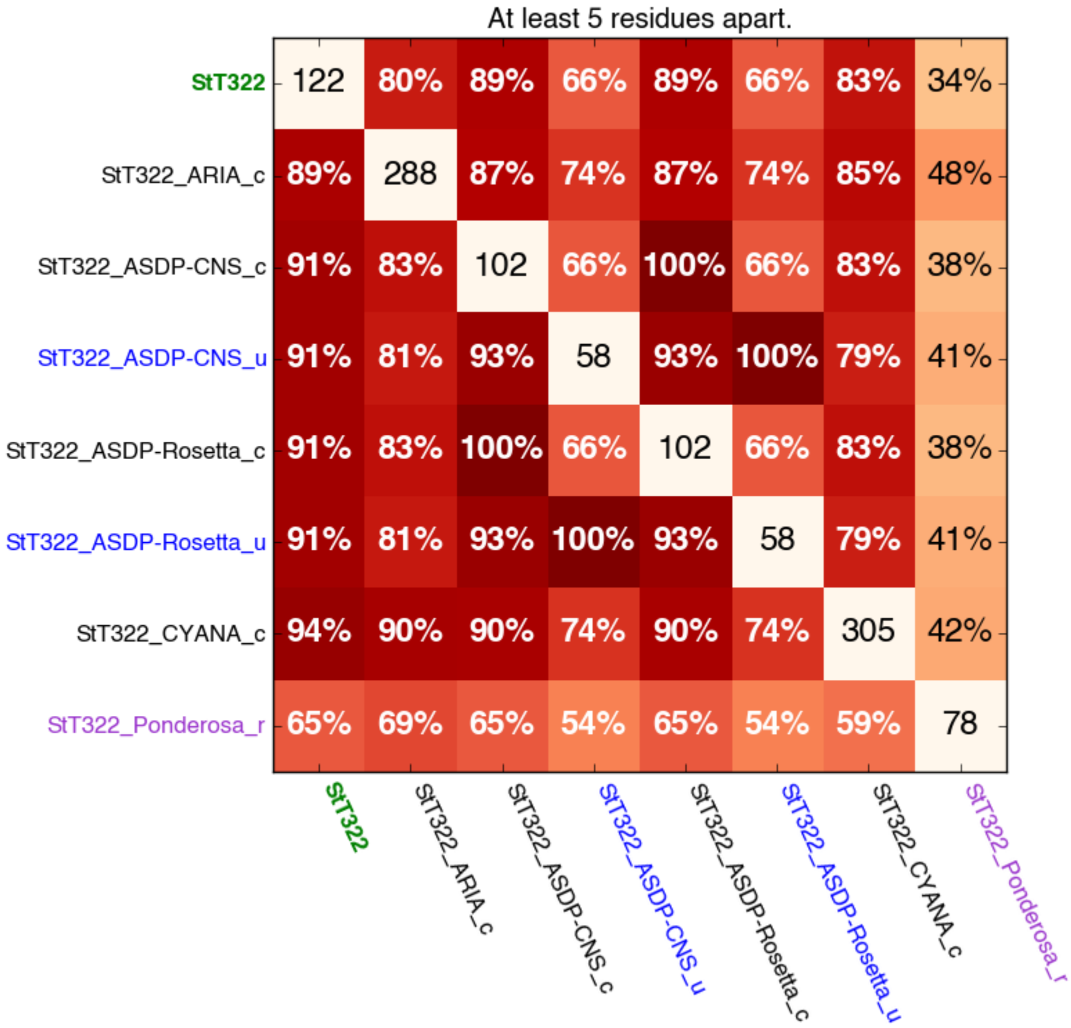


Supplementary Figure 9B. Heatmap of the fractions of overlapping long-range NOE restraints between the StT322 target and entries, determined on the basis of residue.

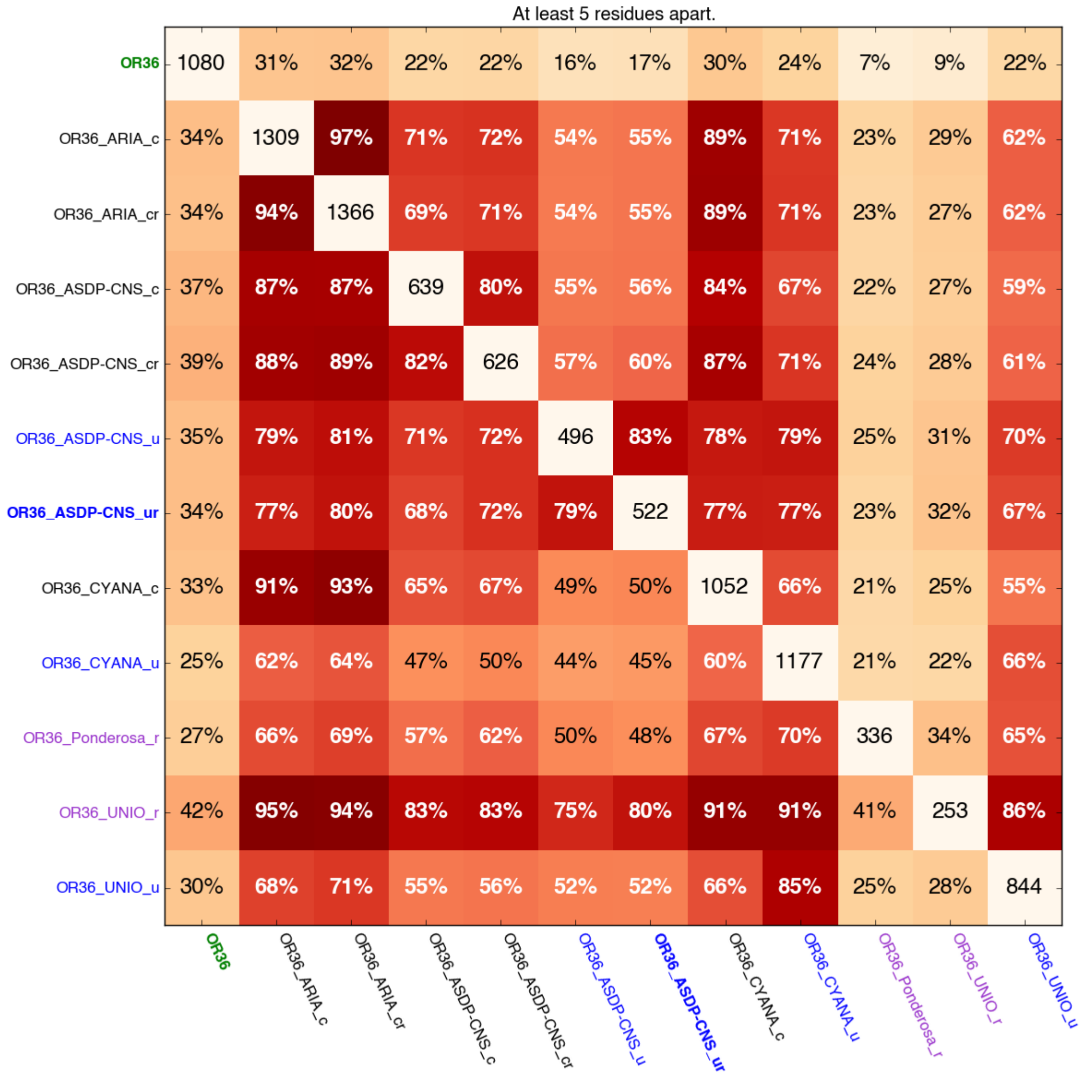


Supplementary Figure 10A. Heatmap of the fractions of overlapping long-range NOE restraints between the OR36 target and entries, determined on the basis of pseudo-atom.

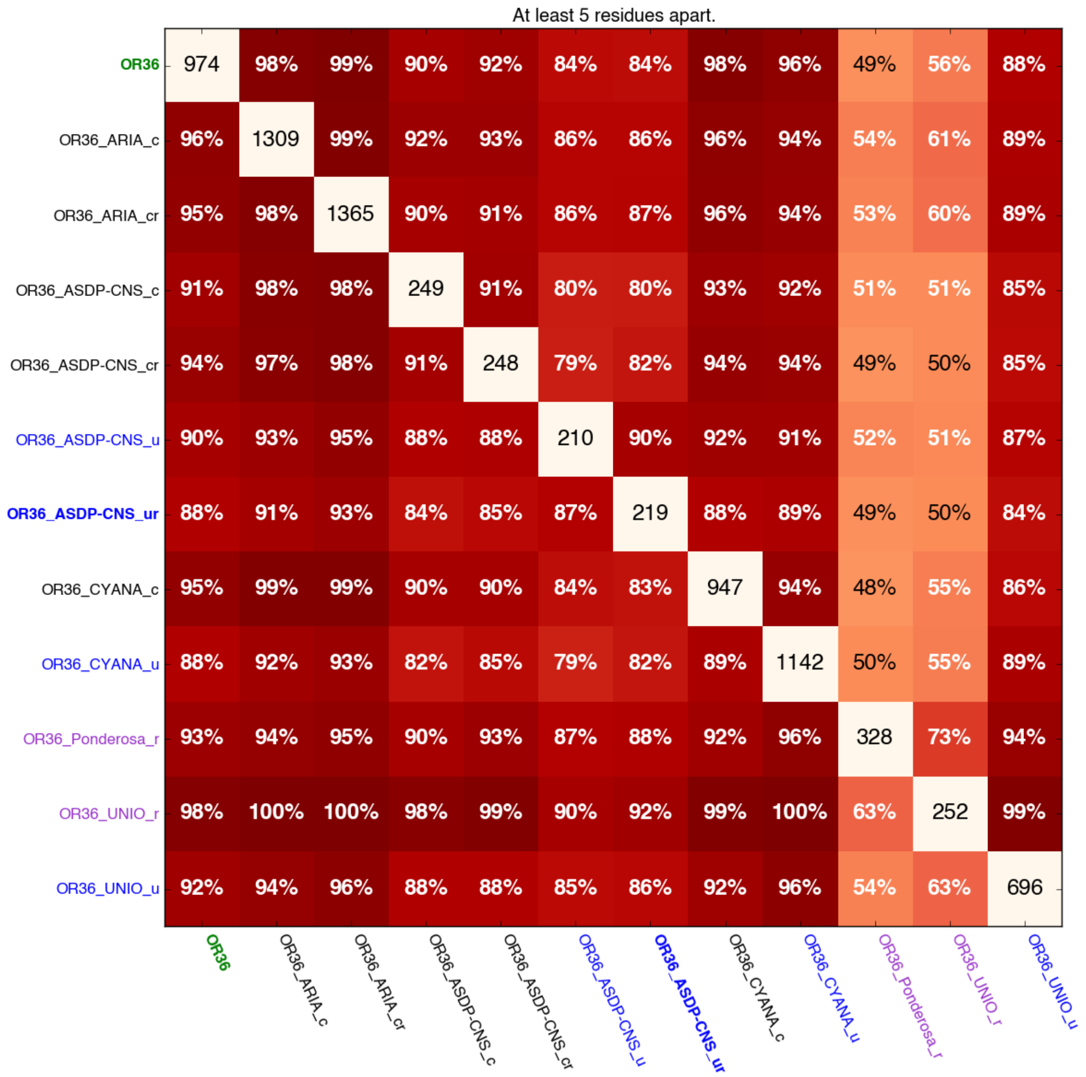


Supplementary Figure 10B. Heatmap of the fractions of overlapping long-range NOE restraints between the OR36 target and entries, determined on the basis of residue.

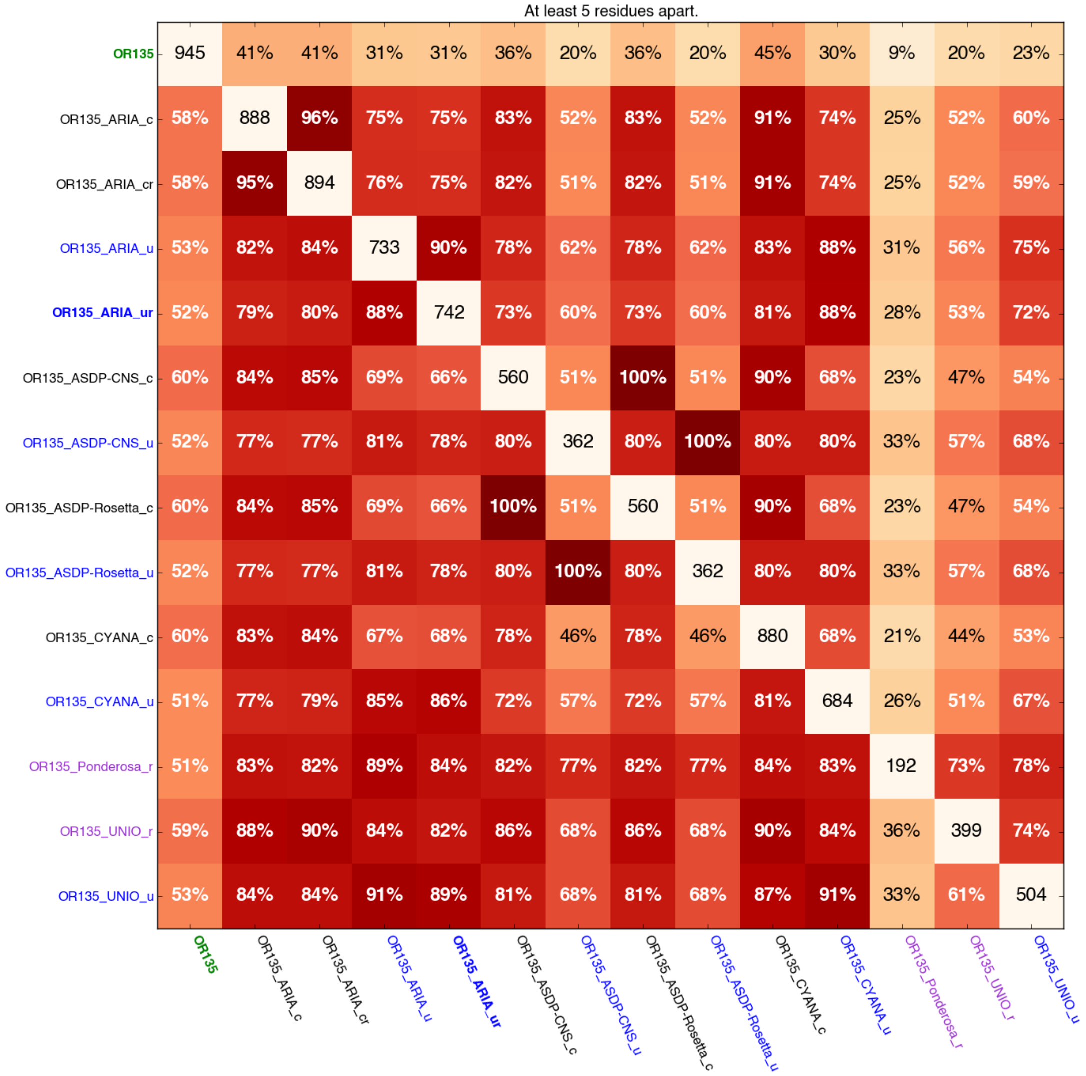


Supplementary Figure 11A. Heatmap of the fractions of overlapping long-range NOE restraints between the OR135 target and entries, determined on the basis of pseudo-atom.

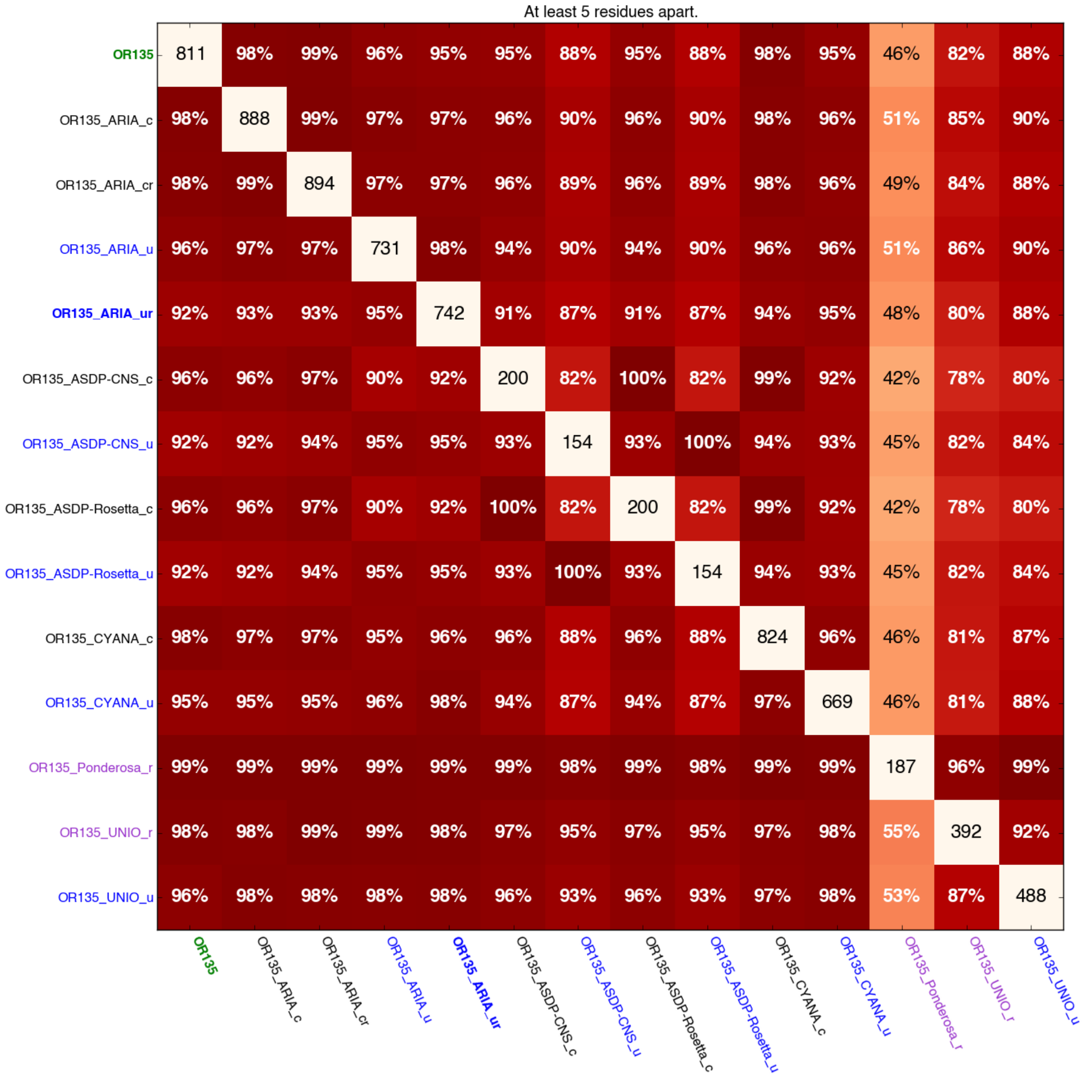


Supplementary Figure 11B. Heatmap of the fractions of overlapping long-range NOE restraints between the OR135 target and entries, determined on the basis of residue.
